# Interfacial Engineering Enables Flexible Composite Film Achieving Ultrahigh Thermal Conductivity and Wave Transparency

**DOI:** 10.1007/s40820-026-02297-3

**Published:** 2026-07-14

**Authors:** Kaiyuan Li, Linhong Li, Guichen Song, Yue Qin, Hanxi Chen, Zujian Zhao, Boren Yang, Yiwei Zhou, Yandong Wang, Rongjie Yang, Maohua Li, Fei Chen, Tao Cai, Cheng-Te Lin, Kazuhito Nishimura, Nan Jiang, Jinhong Yu

**Affiliations:** 1 State Key Laboratory of Advanced Marine Materials, Zhejiang Key Laboratory of Extreme-environmental Material Surfaces and Interfaces, Ningbo Institute of Materials Technology and Engineering, Chinese Academy of Sciences, Ningbo, 315201 People’s Republic of China; 2 School of Materials Science and Chemical Engineering, Ningbo University, Ningbo, 315211 People’s Republic of China; 3 Center of Materials Science and Optoelectronics Engineering, University of Chinese Academy of Sciences, Beijing, 100049 People’s Republic of China; 4 Gansu Engineering Research Center of Eco-Environment Intelligent Networking, College of Electrical Engineering, Northwest Minzu University, Lanzhou, 730000 People’s Republic of China; 5 School of Integrated Circuits, Engineering Research Center for Functional Ceramics MOE, Huazhong University of Science and Technology, Wuhan, 430074 People’s Republic of China

**Keywords:** Hexagonal boron nitride, Surface functionalization, Thermal conductivity, Wave transparency

## Abstract

With the trend toward high-power radio-frequency (RF) electronic devices, flexible thermally conductive composite films are regarded as a key engineering interfacial material to address the critical problem of signal transmission and heat dissipation. However, achieving simultaneous optimization of high thermal conductivity and low loss in the same material remains a significant challenge, particularly in films with high filler loading. Here, a bottom-up regulatory strategy is proposed to address this challenge. Through the synergistic optimization of interface modification design and spatial orientation arrangement of hexagonal boron nitride (BN), the fabricated composite film achieves an exceptional in-plane thermal conductivity of 78.50 W m⁻^1^ K⁻^1^, significantly surpassing that of the film with BN. This remarkable enhancement is primarily attributed to the altered surface properties of the aminated BN (BN-NH_2_). The introduction of amine groups enhances the compatibility with the polymer matrix through electrostatic interactions to facilitate more efficient interfacial thermal transport. Furthermore, the highly oriented spatial arrangement of BN in the film not only preserves the electromagnetic wave transparency (99.94%), but also enhances its breakdown strength. This study underscores the critical role of molecular-level filler modification combined with micro-nanostructural engineering in synergistically strengthening the filler-matrix interface and boosting film thermal transport.

**Supplementary Information:**

The online version contains supplementary material available at 10.1007/s40820-026-02297-3.

## Highlights

- Through synergistic modulation of interface modification and spatial orientation, the scalable fabrication of BN-based thermal management films has been achieved.
- The resulting films exhibit exceptional thermal conductivity reaching 78.50 W m^−1^ K^−1^ and electromagnetic wave transmittance over 99.9%, positioning this work at the forefront of the field.
- This film demonstrates considerable potential for application in interfacial signal integrity and thermal management in radio-frequency electronic devices.

**Supplementary Information:**

The online version contains supplementary material available at 10.1007/s40820-026-02297-3.

## Graphical abstract

**Figure:**
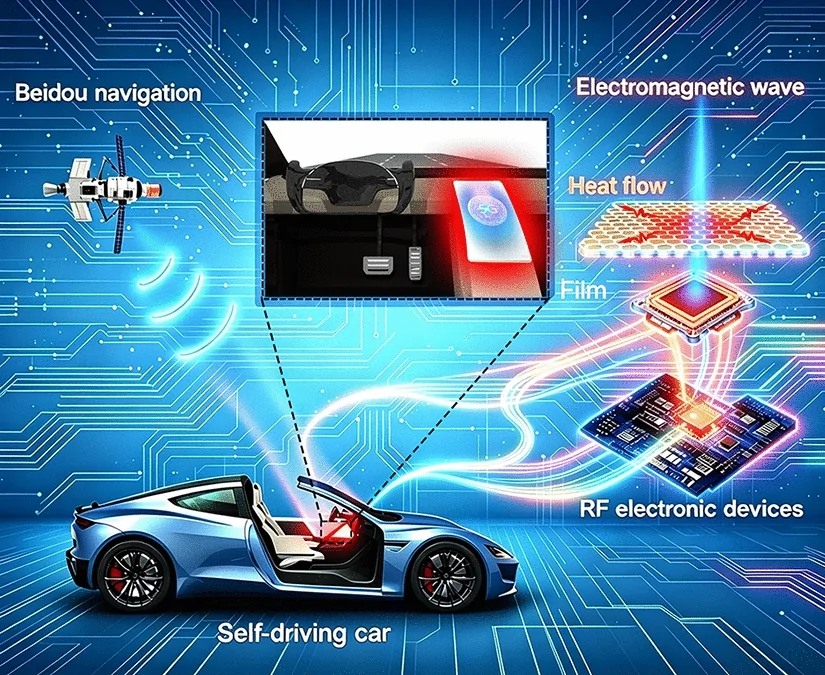

## Introduction

The rapid advancement of high-frequency 5G communication and autonomous driving system has imposed increasingly stringent requirements on the design of thermal management materials for power electronic devices. Particularly for RF signal components, the design of thermal management materials must reconcile often conflicting requirements: achieving exceptional thermal conductivity and mechanical flexibility to dissipate heat, while maintaining ultralow signal interference (typically below 0.5% loss) to ensure unimpeded high-frequency signal transmission [1–4]. To address these challenges, polymer-based composites have been widely adopted for thermal management in RF signal devices due to their functional versatility, scalability, and cost-effectiveness [5–8]. However, the intrinsically low thermal conductivity of polymers poses a significant limitation in meeting the escalating thermal management demands of next-generation high-power devices.

Based on Umklapp phonon-scattering theory, a common strategy for enhancing the thermal conductivity of polymers is the selective incorporation of fillers with highly thermal conductivity into polymer matrix [9, 10]. Therefore, low-dimensional ordered materials [11–13], such as graphene [14–17], carbon fibers [18–20], alumina [21, 22], and hexagonal boron nitride (BN) [23–25], are incorporated into polymer matrix to improve lattice density and structural ordering, thereby enhancing thermal transportation performance. Among these fillers, BN exhibits an ultrahigh theoretical thermal conductivity of 200–2000 W m^−1^ K^−1^, due to its well-defined crystal structure, which constrains the phonon scattering [26–28]. Furthermore, the non-delocalized electron movement between boron and nitrogen atoms results in a wide bandgap, granting BN excellent electrical insulation properties [29–31]. These combined characteristics make BN an ideal candidate for developing novel thermal management materials in RF devices [32–34]. Consequently, designing aligned films that fully utilize the preferred phonon transport direction of BN to achieve high thermal conductivity has attracted considerable research attention. Pioneering studies have demonstrated the effectiveness of constructing well‑oriented BN architectures in boosting thermal conductivity. For instance, by employing a unique ice‑template method combined with hot‑pressing, researchers established a continuous thermal pathway across the BN‑polymer interface, resulting in a film with a high thermal conductivity of 21.3 W m^−1^ K^−1^ [35]. Similarly, Yu et al. utilized the hot‑pressing method to induce highly aligned BN arrangements in thermoplastic polymers, achieving an exceptional in‑plane thermal conductivity up to 73.38 W m^−1^ K^−1^ in the resulting films [36].

However, there exists an inverse coupling between the thermal conductivity performance and mechanical properties of films. Particularly at high filler loadings, BN tends to aggregate rather than disperse uniformly within the polymer matrix, which limits its ability to enhance the thermal transport of the composites. Moreover, the non-uniform dispersion of fillers can severely degrade other critical properties, such as breakdown strength and wave transparency [37]. Therefore, regulating the distribution of BN within the polymer matrix through surface modification has become a key strategy for further improving the heat conduction of films [38, 39]. For instance, Huang et al. modified BNNS with oxalyl chloride to introduce a negative surface charge, which enhanced its dispersion in the matrix via electrostatic interactions, resulting in a film with excellent thermal conductivity and low dielectric loss [40] Similarly, previous works have shown that silane coupling agents can significantly improve the dispersion and compatibility of fillers in the matrix, leading to a notable increase in the thermal conductivity of the composites [41–43]. For example, the incorporation of silane-modified aluminum filler reduced the interfacial thermal resistance between the filler and the matrix, raising the thermal conductivity of composite [44]. Nevertheless, the interaction mechanisms between modifiers and fillers, as well as the surface modification enhances heat conduction in composites, remain poorly understood. Clarifying these mechanisms represents a critical challenge in developing films that simultaneously exhibit high thermal conductivity and low signal transmission loss.

Drawing on the established role of silane coupling agents in regulating nanoscale thermal transport, this work proposes a bottom-up preparation strategy designed to overcome key challenges in filler modification and high interfacial thermal resistance, ultimately yielding films with excellent thermal conductivity and wave‑transparent performance. Firstly, BN was modified with a positive surface charge to enhance its dispersibility within the polymer matrix. Density functional theory (DFT) simulations were employed to identify the grafting sites and interaction mechanisms of the modifier on the BN surface. Subsequently, a combination of doctor-blade casting and vacuum hot-pressing was applied to induce highly oriented in‑plane alignment of BN and to establish strong filler-matrix interfacial bonding. Molecular dynamics (MD) simulations confirmed a marked increase in interfacial heat conduction efficiency for modified BN compared to its unmodified counterpart, indicating that electrostatic interactions between BN-NH_2_ and the acrylic matrix reinforce the interface interaction and enhance overall thermal transport. Through this systematic investigation spanning from nanoscale modification to macroscale structural regulation, a scalable film was successfully fabricated, exhibiting a thermal conductivity reaching 78.50 W m^−1^ K^−1^ and a wave transmittance up to 99.94%. This preparation strategy is characterized by simplicity, high efficiency and scalability, demonstrating promising potential for advanced applications in thermal management of RF signal devices.

## Experimental Section

### Materials

The BN were purchased from Shandong Jonye Advanced Material Co., Ltd. With an average diameter of 20 μm. The NaOH, H_2_O_2_ and γ-Aminopropyltriethoxysilane (APTES) were provided by Shanghai Aladdin Biochemical Technology Co., Ltd. And the acrylic ester (AE) was obtained from Guangzhou Ruishi Biotechnology Co., Ltd..

### Preparation of BN-NH_2_, V-AE/BN Films and V-AE/BN–NH_2_ Films

#### Preparation of BN-NH_2_

Firstly, BN was reacted with a solution with a 7:3 volume ratio of NaOH and H_2_O_2_ at 85 °C for 8 h. This step was designed to remove organic contaminants and increase the hydroxyl density on the BN surface [45]. Upon completion, the BN was thoroughly washed with deionized water and then dried at 60 °C for 1 h to afford hydroxylated BN (BN-OH). Following hydroxylation, BN-OH and APTES were added to the ethanol solution (C_2_H_5_OH: H_2_O = 9:1) and reacted at 85 °C for 8 h [46]. Finally, any physically adsorbed APTES was washed away with ethanol, followed by drying of the powder at 60 °C for 1 h to obtain the final product.

#### Preparation of V-AE/BN-NH_2_ Films

In the initial step, BN-NH_2_ powder was added to the deionized water. The mixture was processed in a SpeedMixer at 3500 rpm for 10 min to fully disperse BN-NH_2_ in deionized water. Subsequently, AE was added to the aqueous suspension of BN-NH_2_ and the mixture was homogenized at the same stirring speed for 5 min. After that, AE/BN-NH_2_ films were prepared by tape casting the resulting slurry. During the tape-casting procedure, the film thickness was controlled between 50 and 70 μm, and the AE/BN-NH_2_ films were dried in an oven at 60 °C. In the next step, the prepared AE/BN-NH_2_ films were placed into a vacuum press for processing. The temperature of the machine was set at 160 °C, with a heating time of 100 s, a vacuum-holding time of 20 s and an applied pressure of 10 t. Finally, V-AE/BN-NH_2_ films can be obtained. For a comprehensive comparison, the preparation of V-AE/BN films were the same as that of V-AE/BN–NH_2_ film, except that the BN was unmodified.

### Material Characterizations

The microstructure and morphology of BN, V-AE/BN, and V-AE/BN-NH_2_ were observed by a field emission scanning electron microscope (SEM, HITACHI, SEM Regulus 8230, Japan). The surface elements and chemical states of the samples were determined by X-ray photoelectron spectroscopy (XPS, Shimadzu Enterprise, AXIS SUPRA +, Japan). The surface chemical composition and structure of the crystals were analyzed using Fourier transform infrared spectroscopy (FTIR, Nicolet 6700, Thermo Fisher Scientific, USA), X-ray diffraction (XRD, D8 Discover/GADDS, Bruker, Germany), 2D Wide-Angle X-ray Diffraction (WAXD, Xeuss 2.0, Xenocs, France), and Raman spectroscopy (HORIBA, LabRAM Odyeeey, France) with a 532 nm laser. The zeta potential of the sample was determined using the Zeta Potential Analyzer (ASAP2460, Micromeritics, USA). The thermal conductivity (K) of the film was calculated using the formula *K* = *α* × *ρ* × *C*_p_, where *α* is the thermal diffusivity, measured by the laser flash method (LFA 467 NanoFlash, Netzsch, Germany),* ρ* is the density, determined by the water displacement method, and *C*_p_ is the specific heat capacity, obtained by differential scanning calorimetry (DSC, PYRIS Diamond, Perkin Elmer, USA). Thermogravimetric analysis (TGA, TG-209 F3, Netzsch, Germany) of the films were carried out under a N_2_ atmosphere. Thermal images were captured by an infrared (IR) camera (Ti400, Fluke, USA). The wave transmission in the 2–18 GHz range was characterized with a vector network analyzer (VNA, AV3672D, Shenzhen Yinjianglong Electronics Co., Ltd., China). The dielectric properties of the films were measured at room temperature using an LCR meter (TH 2838A, Changzhou Tonghui Electronics Co., Ltd., China). The breakdown strength of the composite films was tested using a voltage-withstand testing device (ET2671B, Entai, Nanjing, China).

### Simulation Method

First-principles calculations were carried out using density functional theory as implemented in the CASTEP code [47]. Ultrasoft pseudopotentials and the GGA-PBE exchange–correlation functional were employed. A plane-wave cutoff energy of 500 eV and a Monkhorst–Pack k-point mesh of 2 × 2 × 1 were used. A vacuum spacing of 20 Å was applied to avoid interlayer interactions. All structures were fully relaxed until forces were below 0.01 eV Å^−1^. Adsorption and binding energies were calculated based on total energy differences. Charge density differences were obtained to analyze interfacial charge transfer.

All molecular dynamics simulations were performed using the Large-scale Atomic/Molecular Massively Parallel Simulator. The polymer matrix was modeled using a COMPASS-type Class II force field with a 9–6 Lennard–Jones potential for non-bonded interactions, while the monolayer BN was described using the Tersoff potential. A hybrid potential scheme was employed to describe the polymer/BN composite system, and the interfacial interactions were treated using the same Class II force field. Prior to thermal transport simulations, the system was equilibrated at 350 K for 1,000,000 fs under the NVT ensemble, with atoms within 20 Å at the top and bottom fixed. Non-equilibrium molecular dynamics simulations were then conducted under the NVE ensemble by applying equal heating and cooling power (0.1 kcal mol^−1^ fs^−1^) to two regions of 10 Å thickness. After reaching a steady-state temperature gradient, the temperature profile was obtained by time-averaging over an additional 1,000,000 fs. More DFT and MD simulations analysis details were found in Supplementary Materials.

## Results and Discussion

### Synthesis, Characterization, and Reaction Mechanism of BN-NH_2_

**Fig. 1:**
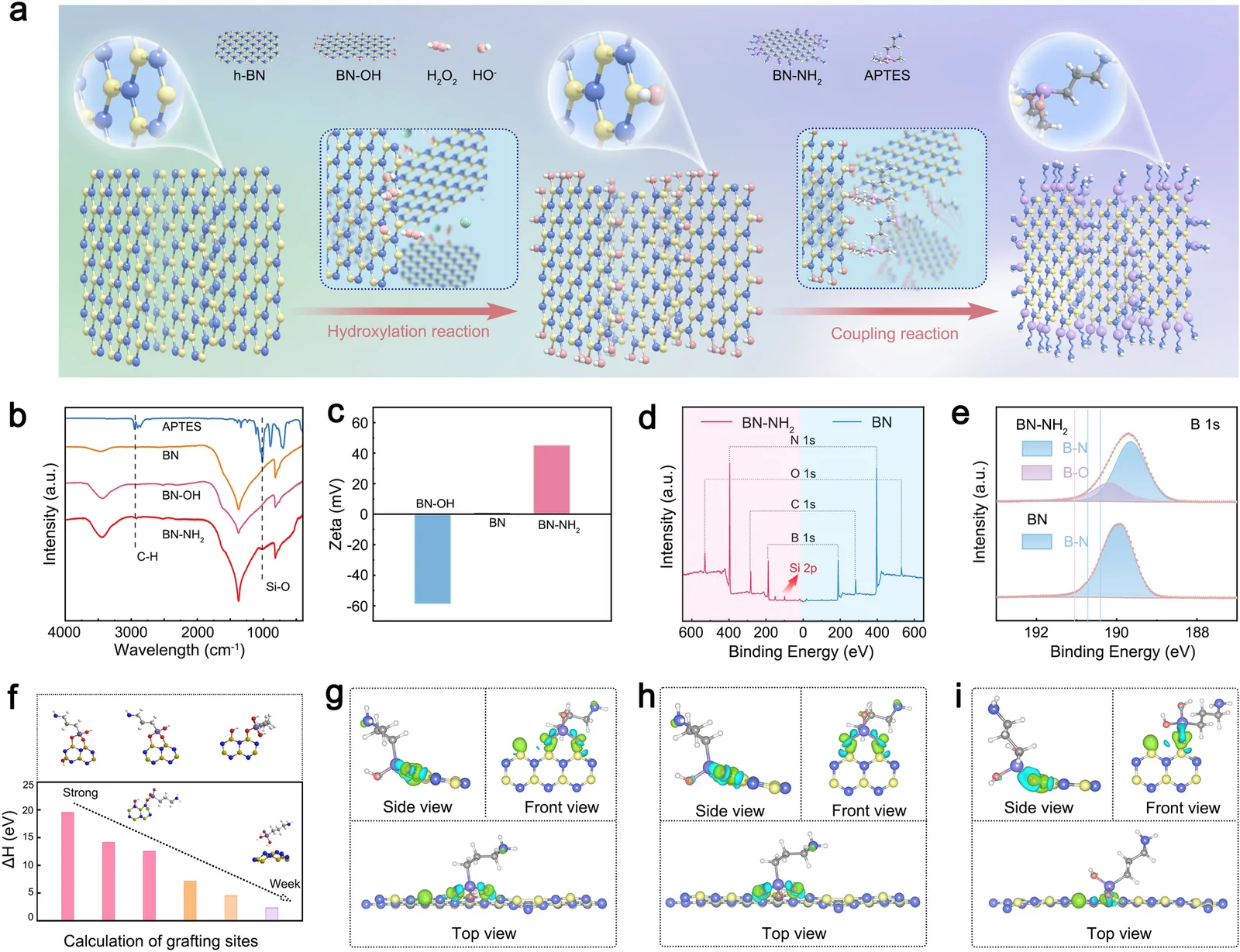
Synthesis, characterization, and reaction mechanism of BN-NH_2_. **a** Schematic diagram of amino modification of BN powder. **b** FTIR spectra of APTES, BN, BN-OH, and BN-NH_2_. **c** Zeta potentials of BN, BN-OH, and BN-NH_2_. **d** XPS spectra of BN and BN-NH_2_. **e** B 1*s* spectra of BN and BN-NH_2_. **f** Adsorption energies of different grafting positions calculated by DFT simulation. **g-i** Charge density difference of different grafting positions

In order to reduce the steric hindrance effect caused by high BN content and enhance the interfacial interaction between the filler and the polymer matrix, active functional groups compatible with the polymer are introduced onto the surface of BN. Figure 1a schematically presents the surface functionalization strategy of BN powder. Distinguished from pristine BN (Fig. S1), there is an obvious absorption peak at approximately 3400 cm^−1^, corresponding to the O–H stretching vibration (Fig. 1b) [48, 49]. Meanwhile, the characteristic Si–O peak at 1050 cm^−1^ and the distinct C–H stretching vibration peak at 2920 and 2850 cm^−1^ of APTES can be clearly observed [50] indicating that the surface of BN contains a large number of activated functional groups.

Figure 1c displays the Zeta potential measurements of three distinct BN powders. The pristine BN exhibits near-neutral charge, indicative of its electrically neutral surface. After alkaline etching, the BN-OH acquires a pronounced negative charge, attributed to the introduction of abundant surface hydroxyl groups, which will promote subsequent silane hydrolysis and grafting. Notably, the negatively charged BN transform into a positively charged state after treatment with APTES, due to the introduction of a large amount of -NH_2_ groups on its surface. XPS analysis was performed to investigate the chemical composition and structure of BN-NH_2_, as shown in Fig. 1d. The survey spectrum of BN-NH_2_ reveals a distinct Si 1*s* peak, and the intensities of its C 1*s* and O 1*s* peaks are significantly enhanced relative to those of pristine BN. And the high-resolution B 1*s* spectrum (Fig. 1e) reveals a new peak at 190.5 eV, corresponding to a Si–O–B bond, while the N 1*s* spectrum (Fig. S2) shows a characteristic peak at 397.6 eV of N–H, together confirming successful surface amination [51, 52]. Additionally, XRD patterns remain nearly identical before and after modification (Fig. S3), indicating that the chemical modification process does not alter or damage the crystalline structure of BN [53].

**Fig. 2:**
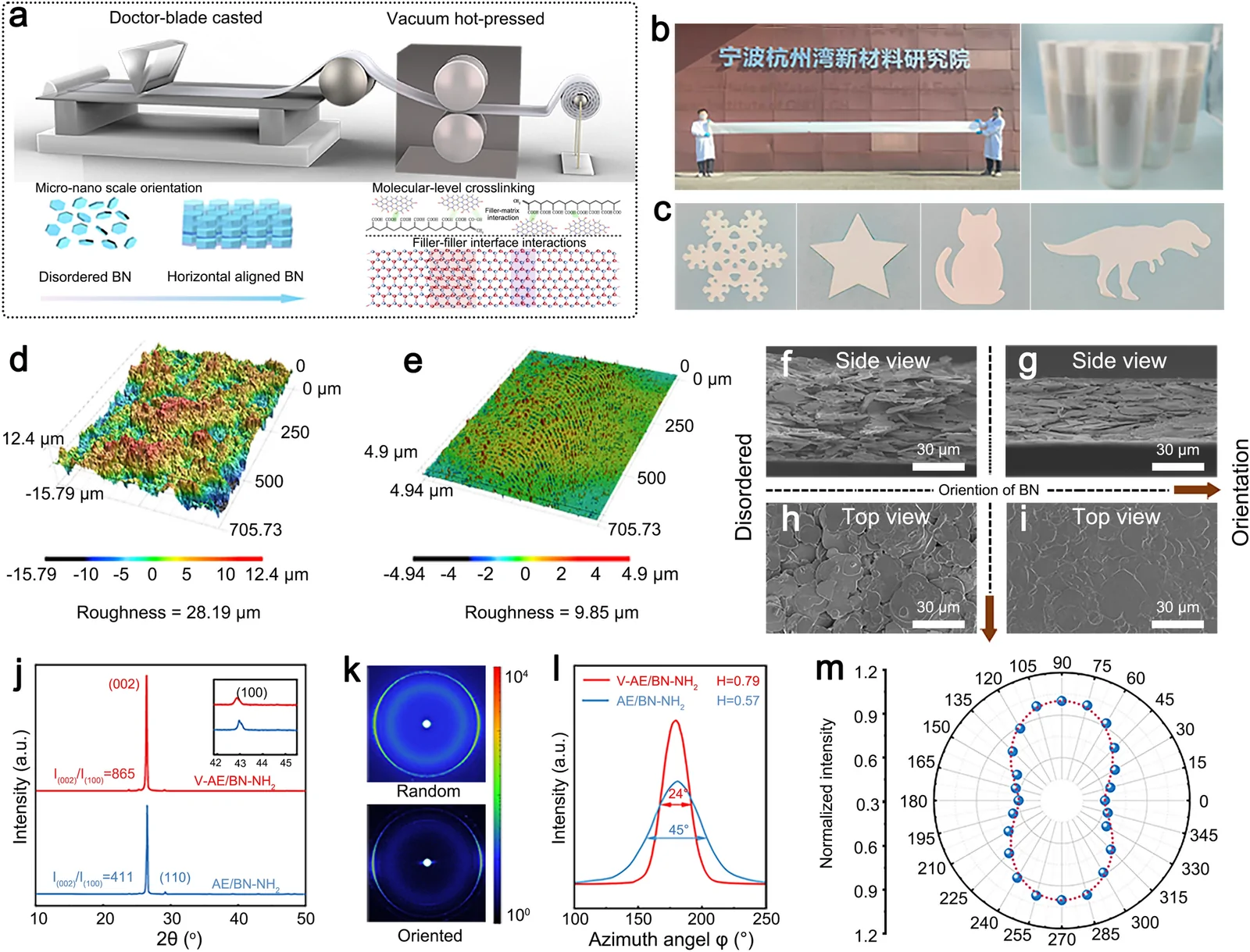
Preparation and structural design of the films. **a** Demonstration of materials preparation process mechanism. **b** Schematic illustrating the large-scale films preparation. **c** Optical image displaying cuttability of films. The 3D Surface morphology of **d** AE/BN-NH_2_ film and **e** V-AE/BN-NH_2_ film. The cross-section SEM micrograph **f** AE/BN-NH_2_ film and **g** V-AE/BN-NH_2_ film. The surface SEM micrograph of **h** AE/BN-NH_2_ film and **i** V-AE/BN-NH_2_ film. **j** XRD patterns of AE/BN-NH_2_ and V-AE/BN-NH_2_ films. **k** 2D-WAXD patterns of AE/BN-NH_2_ and V-AE/BN-NH_2_ films. **l** Azimuth angle and Herman’s orientation parameter. **m** A1 g band intensity, IA1 g (θ), as a function of the angle θ between the cross section of the V-AE/BN-NH_2_ film and electric field vector of the incident laser

To identify the preferential bonding sites of APTES, the DFT calculations (the details can be found in Supplementary Text S1.4) were performed to evaluate the adsorption energy between BN and APTES (Fig. 1f) [54]. Notably, for adsorption at the BN edge, structural relaxation leads to a spontaneous dissociation of the hydroxyl group in APTES without imposing any reaction pathway or transition-state constraint. Specifically, the H atom from the –OH group transfers to an edge B atom, forming a B–H bond, while the remaining –O fragment binds to a neighboring B atom, resulting in a dissociative chemisorption configuration. The emergence of this dissociated structure upon geometry optimization indicates that it corresponds to a thermodynamically stable adsorption state. Pronounced charge redistribution is observed at the B–O and B–H bonding regions (Figs. 1g-i), further supporting the formation of strong chemical interactions at the BN edge (Fig. S4). It should be noted that no kinetic barriers were evaluated in this work, and the present results focus on the relative energetic stability of different adsorption configurations.

### Preparation and Structural Design of the Films

To enhance the interfacial strength between polymer matrix and BN, it is essential to optimize the stacking density of BN. This study employs a sequential two-step procedure integrating doctor-blade casting and vacuum hot pressing to achieve superior orientation structure of BN (Fig. 2a). Firstly, the shear forces generated during the doctor-blade deposition process (the details can be found in Supplementary Text S1.5) are utilized to promote the uniaxial alignment of the BN along the horizontal direction (Fig. S5) [55]. Subsequently, hot pressing under vacuum is conducted to promote interfacial bonding between BN and the polymer matrix, which effectively reduces interfacial voids of filler and the polymer matrix while further enhancing the alignment degree of BN. This well-engineered continuous fabrication process enables the scalable manufacturing of the films, and a representative sample of the resulting film is displayed in Fig. 2b. Even at a high BN loading, the film also can be cut, folded and rolled without obvious damage, demonstrating excellent mechanical properties (Figs. 2c and S6).

Figure 2d, e presents the three-dimensional surface morphology of the film before and after hot-pressing, showing a sharp decrease in surface roughness from 28.19 to 9.85 μm. This change originates from the altered spatial arrangement of BN. Accordingly, compared to the AE/BN-NH_2_ film (Fig. 2f), the internal structure of the V-AE/BN-NH_2_ film is significantly optimized as shown in Fig. 2g. It indicates that the mismatched structure of BN generated during the blade coating process has been transformed into a long-range ordered parallel arrangement structure through the application of horizontal pressure. Furthermore, it can be found that the integrated welding of BN was realized due to the viscoplastic behavior of acrylic ester matrix at high temperature, which transformed the film surface from a dispersed porous structure into a flat and compact state (Fig. 2h, i). Meanwhile, given that different orientations of BN spatial arrangement produce distinct diffraction patterns, the XRD was employed to assess the BN alignment (Fig. 2j). The presence of sharp diffraction peaks at 26.4°, 29.1°, and 42.8° in the normalized XRD pattern is attributed to diffraction from the (002), (100), and (110) planes of BN [56]. The extent of horizontal orientation was evaluated by the intensity ratio of I _(002)_ to I _(100)_, with a higher value denoting a better-aligned structure.

Figure 2k compares the 2D wide-angle X-ray diffraction (2D-WAXD) patterns of the BN film before and after hot-pressing. It can be seen the pattern of the randomly oriented sample displays isotropic concentric rings, whereas the hot-pressed V-AE/BN-NH_2_ film exhibits symmetrical arcs, indicative of a highly anisotropic BN arrangement. Additionally, the azimuthal integration of the 2D-WAXD pattern (Fig. 2l) was used to determine the orientation degree (ΔS) and the Herman orientation parameter **h** for the film [57] These parameters were quantified using Eqs. 1 and 2:

1 $$\Delta S={\lambda}_{1}-{\lambda}_{2}=\sqrt{{({S}_{11}-{S}_{12})}^{2}+4{{S}_{12}}^{2}}$$

2 $$H=\frac{3}{2}\frac{{\int}_{0}^{\frac{\pi }{2}}d\Psi{\sin}\Psi I(\Psi){{\cos}}^{2}\Psi}{{\int}_{0}^{\frac{\pi }{2}}d\Psi{\sin}\Psi I(\Psi)}-\frac{1}{2}$$

A detailed account of the calculations is given in Supplementary Texts S1.6. Furthermore, based on the position of the Raman characteristic peak of BN in the films, the peak intensity at 1361 cm^-1^ (Fig. S7) was selected for normalization to generate a polarized Raman polar plot [58]. The plot exhibits a double-lobed shape and is fitted in Fig. 2m (The specific details shown in Supplementary Text S1.7). The high correlation between the experimental data points and the fitted curve highlights the excellent uniaxial orientation of the BN films.

### Thermal Properties and Heat Conduction Mechanism of V-AE/BN-NH_2_ Film

**Fig. 3:**
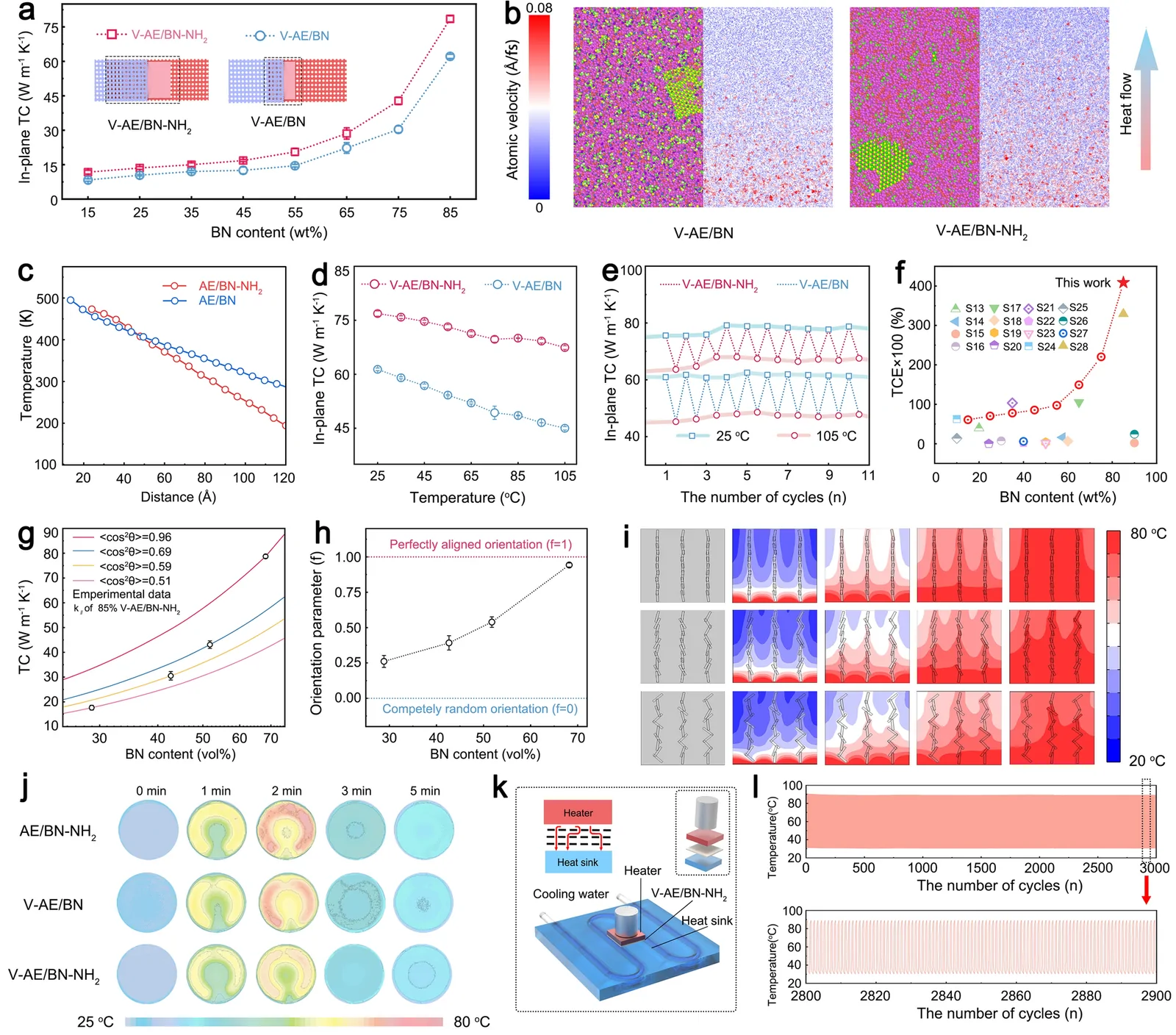
Thermal properties and heat conduction mechanism of V-AE/BN-NH_2_ film. **a** Thermal conductivity of V-AE/BN-NH_2_ and V-AE/BN films. **b** Molecular dynamics simulation of the atomic velocity. **c** Temperature gradients of the AE/BN and AE/BN-NH_2_ films. **d** Thermal conductivity of V-AE/BN-NH_2_ and V-AE/BN films at different temperatures. **e** Thermal conductivity of V-AE/BN-NH_2_ and V-AE/BN films over 10 heating and cooling cycles. **f** Comparison of thermal conductivity enhancement of V-AE/BN-NH_2_ film with different BN contents prepared by four methods. **g** Fitting of experimental *K*_∥_ for V-AE/BN-NH_2_ films based on foam theory. **h** Orientation parameter of V-AE/BN-NH_2_ films as a function of BN content. **i** Simulated transient temperature distribution of films with different orientation parameters. **j** Infrared images of AE/BN, V-AE/BN, and V-AE/BN-NH_2_ films. **k** Schematic diagram of the thermal shock resistance experimental setup. **l** Thermal shock stability of V-AE/BN-NH_2_ film in cyclic heating and cooling tests

Owing to the highly aligned orientation of BN in the polymer matrix, the film endows exceptional in-plane thermal conductivity. As shown in Fig. 3a and Supplementary Table S3.2, both the thermal diffusivity (Fig. S8a) and thermal conductivity of the films exhibited an increase with increasing BN content. Notably, the V-AE/BN-NH_2_ film demonstrates superior in-plane thermal diffusivity and thermal conductivity compared with the V-AE/BN counterpart, achieving a remarkable thermal conductivity of 78.50 W m^−1^ K^−1^. It is postulated that the enhanced thermal conductivity of the V-AE/BN-NH_2_ film may be attributed to strengthened interfacial interactions between the BN-NH_2_ and the acrylic matrix through electrostatic interaction, which reduced phonon scattering at the filler-matrix interface thereby facilitating more efficient heat conduction.

In order to verify this hypothesis, the densities of both the films were tested under the same preparation process (Fig. S8b). The V-AE/BN-NH_2_ film consistently exhibited a higher density than that of the V-AE/BN film, suggesting that the aminated BN filler achieves denser packing and lower porosity in the matrix. Furthermore, the MD simulations were employed to evaluate the interfacial heat conduction performance between the filler and the matrix. Figure 3b clearly reveals that the thermal response of V-AE/BN-NH_2_ is superior to that of V-AE/BN. It is attributed to the inherent chemical inertness of the pristine BN surface, which introduces significant interfacial thermal resistance between BN and AE, thereby severely impeding phonon propagation and resulting in poorer thermal transport. In contrast, the BN-NH_2_ exhibits strong electrostatic interactions with AE, which enhances the interfacial bonding strength between the filler and polymer matrix, and consequently leads to a notably improved thermal response in the V-AE/BN-NH_2_ film. The temperature distribution presented in Fig. 3c further confirms that the surface amination of BN is beneficial to enhancing the heat conduction performance of film. Based on the temperature profiles, the interfacial thermal conductivities of V-AE/BN and V-AE/BN-NH_2_ were calculated as 1.04 and 1.49 W m^−1^ K^−1^. A detailed account of the simulation is given in Supplementary Text S1.8.

To investigate the influence of ambient temperature on the thermal transport properties, the thermal conductivity variation with temperature and the cyclic thermal stability of the films were examined. The thermal diffusivity and thermal conductivity decrease with rising temperature (Figs. 3d and S8c), a trend consistent with the Umklapp phonon-scattering mechanism. As temperature rises, the average phonon mean free path decreases and phonon-scattering intensifies, leading to the observed reduction in both thermal diffusivity and thermal conductivity of the films. Meanwhile, the V-AE/BN-NH_2_ and V-AE/BN films were subjected to ten heating and cooling cycles over the temperature range of 25 to 105 °C (Figs. 3e and S8d). It can be clearly seen that the V-AE/BN-NH_2_ film exhibits minimal thermal conductivity variation, highlighting its stable and reliable heat conduction performance.

Figure 3f presents a comparison of the thermal conductivity enhancement (TCE) between our films and previously reported BN-based films (Supplementary Table S3.3). Notably, the TCE of the V-AE/BN-NH_2_ film increases rapidly and surpasses all recently reported values when the BN content exceeds 65 wt%. To comprehensively evaluate the thermal enhancement effect of the highly aligned BN orientation in the polymer matrix and to elucidate the underlying mechanism, the metal foam theory was employed. According to the following Equation [59]:

3 $${K}_{\parallel }=\langle {{\cos}}^{2}\theta \rangle {V}_{BN}{K}_{strut} +{V}_{AE}{K}_{AE}+{V}_{air}{K}_{air}$$

4 $$f=\frac{3\langle {{\cos}}^{2}\theta \rangle -1}{2}$$

A detailed account of the calculations is given in Supplementary Text S1.9. In the given equations, θ is the angle between the BN struts and the heat conduction direction, and ⟨cos^2^θ⟩ denotes the average over all struts. *V*_BN_, *V*_AE_ and *V*_air_ denote the volume fractions of BN (Fig. S9), the acrylic matrix and the entrapped air, respectively. Correspondingly, *K*_strut_, *K*_AE_, and *K*_air_ are the thermal conductivities of a single BN strut (120 W m^−1^ K^−1^), the acrylic matrix (0.19 W m^−1^ K^−1^), and air (0.026 W m^−1^ K^−1^), respectively. Figure 3g presents the fitting of the experimental data to Eq. 3 using different values of ⟨cos^2^θ⟩. The results demonstrate that the set value of ⟨cos^2^θ⟩ gradually increases as the BN content increases to accurately match *K*_∥_, indicating that the increase in BN content leads to an alteration in the degree of orientation within the confined space. Based on this result, the calculated orientation parameter **f** exhibits a distinct increasing trend (Fig. 3h), which is consistent with the mechanism whereby stress-induced orientation adjusts the spatial distribution of BN in the polymer matrix (Fig. S10). When the BN content reaches 68.2 vol%, the value of **f** attains 0.94. This corresponds to an average angle of merely 14.1° between the BN fillers and the heat conduction direction, confirming the formation of a highly ordered orientation within the AE matrix. Subsequently, finite element simulations were performed to analyze the influence of BN orientation angle on thermal response behavior. As shown in Figs. 3i and S11, the simulated temperature gradient between the bottom and top of the heat source markedly decreases with increasing BN alignment, demonstrating that a highly oriented BN structure effectively promotes directional heat conduction in the polymer matrix.

The circular heating test device was built to further evaluate the in-plane heat conduction performance of the film (Fig. S12a), and the temperature evolution was captured using an infrared camera during heating process. As shown in Fig. 3j, the V-AE/BN-NH_2_ film exhibits a more uniform temperature distribution of the thermal images compared with the V-AE/BN and AE/BN-NH_2_ films. Moreover, the temperature difference (Δ*T*) between the center and the edge of the V-AE/BN-NH_2_ film is 10.96 °C, which is significantly lower than 11.72 °C of the V-AE/BN film and 14.97 °C of the AE/BN film (Fig. S12b), indicating the in-plane thermal transport performance is superior to that of another two films. Furthermore, a thermal shock testing system was established to evaluate the long-term stability of the V-AE/BN-NH_2_ film (Fig. 3k). During testing, the samples were tightly sealed between the heat sink and the heater. A cooling system circulating constant-temperature water at 25 °C was employed to maintain the heat sink temperature, while calibrated thermocouples recorded the temperature evolution over time. Figure 3l illustrates that the temperature of the V-AE/BN-NH_2_ film remains nearly constant under repeated thermal shocks induced by cyclic heating and cooling, indicating an outstanding thermal shock stability.

### Application of V-AE/BN-NH_2_ Film Based on the RF Devices

**Fig. 4:**
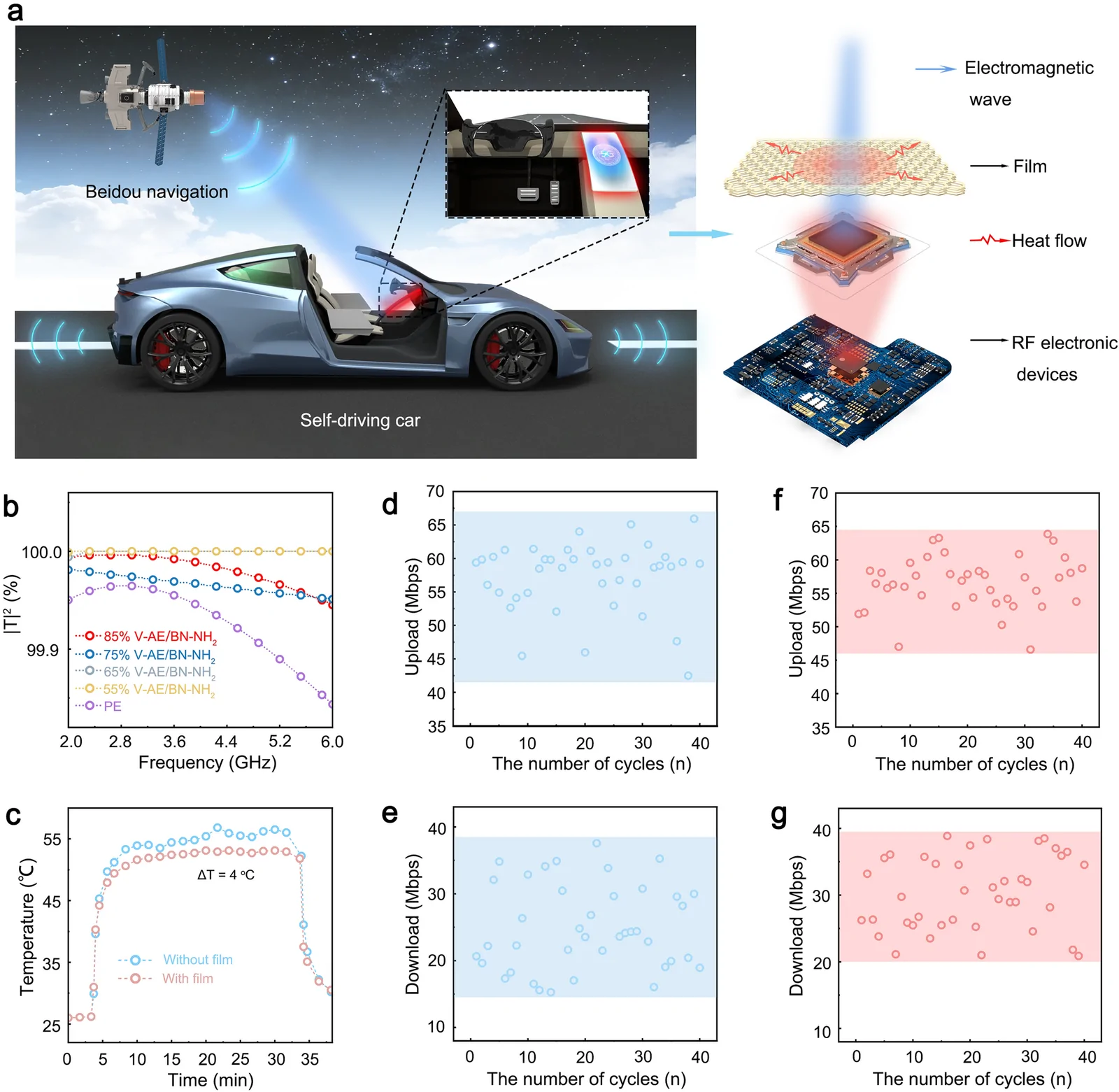
Application of V-AE/BN-NH_2_ film based on the RF devices. **a** Schematic illustration of V-AE/BN-NH_2_ film used in RF electronic devices era. **b** Wave-transparent performance characterization of the V-AE/BN-NH_2_ and PE films. **c** Temperature variation of the router during operation. **d** Upload speed in actual tests without film. **e** Download speed in actual tests without film. **f** Upload speed in actual tests covered with V-AE/BN-NH_2_ film. **g** Download speed in actual tests covered with V-AE/BN-NH_2_ film

Figure 4a illustrates the demand for materials with both high thermal conductivity and excellent wave transmission properties for RF electronic devices in the intelligent driving system of new energy vehicles. During high-frequency data recognition and information processing, electronic devices are prone to significant single point heat concentration. Substantial heat accumulation can severely compromise the signal transmission efficiency and stability of RF devices, thereby compromising the safety performance of the entire intelligent driving system. Currently, commercial polyethylene (PE) films are widely employed as protective insulating materials. However, their relatively low thermal conductivity renders them inadequate in addressing the localized heat generated during RF device operation. Therefore, the thermal management films with both high thermal conductivity and excellent wave transmission properties are regarded as critical materials to support the sustainable development of future intelligent driving technologies.

As shown in Fig. 4b, the V-AE/BN-NH_2_ film exhibits an exceptionally high wave transmittance of 99.94% with negligible signal loss across the 2.0–6.0 GHz frequency range. This performance surpasses that of commercial PE film, demonstrating the superior wave-transparent properties. Furthermore, the V-AE/BN-NH_2_ film possesses a high breakdown strength (direct current) exceeding 20 kV mm^−1^, indicating excellent electrical insulation (Fig. S13a). Moreover, the film shows low dielectric constant and dielectric loss, which effectively prevents heat generation due to energy loss when used in frequency-conversion devices (Fig. S13b). In the frequency range of 10^3^–10^5^ Hz, V-AE/BN and V-AE/BN-NH_2_ films had a low dielectric constant of 3.8–4.3, and the dielectric loss values were less than 0.07. Moreover, a comparison with previous literature on dielectric and wave transmission properties is shown in Supplementary Table S3.5.

To evaluate the practical effectiveness of the V-AE/BN-NH_2_ film in thermal management and wave transmission for RF electronic devices, a thermal management test system based on router signal transmission was constructed (Details shown in Fig.S14). In conventional devices, electronic components in communication equipment are typically left uncovered to ensure unimpeded signal transmission and reception. However, a large amount of heat will be accumulated as the signal transmission power of electronic devices increases, leading to a reduction in signal transmission stability and posing a risk of device failure. In this work, we employed V-AE/BN-NH_2_ film to address the challenges associated with efficient thermal management and electromagnetic wave transmission in communication systems. As illustrated in Fig. 4c, after coating with the V-AE/BN-NH_2_ film, the core temperature of electronic components on a fully loaded router circuit board decreased by approximately 4 °C compared to that of a directly exposed board under the same operating conditions. Moreover, the operating temperature became more stable, demonstrating the excellent practical thermal management capability of the film. Furthermore, signal transmission tests based on a 5G router revealed that the exposed router (Fig. 4d, e) exhibited significant fluctuations in upload and download speeds due to substantial temperature rise and variation during operation. In contrast, the router covered with the V-AE/BN-NH_2_ film (Fig. 4f, g) demonstrated noticeably stabilized upload and download speeds with a narrower confidence interval (Fig. S15). The test results indicate that the film did not interfere with signal transmission and reception, while effectively mitigating the risk of signal fluctuations caused by chip overheating through efficient thermal management of the core electronic components.

## Conclusion

This work comprehensively investigates the significant potential of interfacial modification design and spatial orientation regulation for enhancing the thermal conductivity of BN-based films. The grafting sites and bonding configurations of silane coupling agents on BN surfaces are first examined in detail. Subsequently, an interface design based on electrostatic interactions between the filler and the matrix is implemented, which effectively reduces the overall thermal resistance of the film. Detailed molecular dynamics simulations further confirm a substantial improvement in interfacial heat conduction efficiency. Due to the dual regulation of molecular-level interfacial design and micro-nanoscale orientation structure, the thermal conductivity of the film is significantly increased to 78.50 W m^−1^ K^−1^, establishing a new record in this field. Simultaneously, the film not only possesses superior breakdown strength, but also demonstrates a microwave transmittance exceeding 99.94%. These outstanding characteristics make the film a highly promising candidate for engineering interfacial thermal management in RF electronic devices, paving the way for next-generation high-power applications.

## Supplementary Information

Below is the link to the electronic supplementary material.

Supplementary file1 (DOCX 3220 KB)
