# Supplementary material for: Interfacial Engineering Enables Flexible Composite Film Achieving Ultrahigh Thermal Conductivity and Wave Transparency
Source: Nanomicro Lett. 2026 Jul 14;18:439. doi: 10.1007/s40820-026-02297-3 (PMC13369072; doi:10.1007/s40820-026-02297-3)
Supplement: Supplementary file 1 — Supplementary file1 (DOCX 3220 KB) [file 40820_2026_2297_MOESM1_ESM.docx]

# Supporting Information for

**Interfacial Engineering Enables Flexible Composite Film Achieving Ultrahigh Thermal Conductivity and Wave Transparency**

Kaiyuan Li^1#^, Linhong Li ^2#^*, Guichen Song ^4^*, Yue Qin ^2^, Hanxi Chen ^2, 3^, Zujian Zhao ^1^, Boren Yang ^1^, Yiwei Zhou ^2^, Yandong Wang ^2^, Rongjie Yang ^2, 3^, Maohua Li ^2^, Fei Chen ^5^, Tao Cai ^2, 3^, Cheng-Te Lin ^2, 3^, Kazuhito Nishimura ^2, 3^, Nan Jiang ^2, 3^, Jinhong Yu ^2, 3^*

^1^ School of Materials Science and Chemical Engineering, Ningbo University, Ningbo 315211, P. R. China

^2^ State Key Laboratory of Advanced Marine Materials, Zhejiang Key Laboratory of Extreme-environmental Material Surfaces and Interfaces, Ningbo Institute of Materials Technology and Engineering, Chinese Academy of Sciences, Ningbo 315201, P. R. China

^3^ Center of Materials Science and Optoelectronics Engineering, University of Chinese Academy of Sciences, Beijing 100049, P. R. China

^4^ Gansu Engineering Research Center of Eco-Environment Intelligent Networking, College of Electrical Engineering, Northwest Minzu University, Lanzhou 730000, P. R. China

^5^ School of Integrated Circuits, Engineering Research Center for Functional Ceramics MOE, Huazhong University of Science and Technology, Wuhan 430074, P. R. China

*^#^* Kaiyuan Li and Linhong Li are co-first authors and contributed equally to this work.

*Corresponding authors. E-mail: [lilinhong@nimte.ac.cn](mailto:lilinhong@nimte.ac.cn) (Linhong Li); [songgch@xbmu.edu.cn](mailto:songgch@xbmu.edu.cn) (Guichen Song); [yujinhong@nimte.ac.cn](mailto:yujinhong@nimte.ac.cn) (Jinhong Yu)

**S1 Supplementary Texts**

***S1.1 Materials***

The BN were purchased from Shandong Jonye Advanced Material Co., Ltd. With an average diameter of 20 μm. The NaOH, H_2_O_2_ and γ-Aminopropyltriethoxysilane (APTES) were provided by Shanghai Aladdin Biochemical Technology Co., Ltd. And the acrylic ester (AE) was obtained from Guangzhou Ruishi Biotechnology Co., Ltd..

***S1.2 Preparation of BN-NH_2_***

Firstly, BN was reacted with a solution with a 7:3 volume ratio of NaOH and H_2_O_2_ at 85 °C for 8 h. This step was designed to remove organic contaminants and increase the hydroxyl density on the BN surface. Upon completion, the BN was thoroughly washed with deionized water and then dried at 60 °C for 1 h to afford hydroxylated BN (BN-OH). Following hydroxylation, BN-OH and APTES were added to the ethanol solution (C_2_H_5_OH : H_2_O=9:1) and reacted at 85°C for 8 h. Finally, any physically adsorbed APTES was washed away with ethanol, followed by drying of the powder at 60 °C for 1 h to obtain the final product.

***S1.3 Preparation of V-AE/BN-NH_2_ Films***

In the initial step, BN-NH_2_ powder was added to the deionized water. The mixture was processed in a SpeedMixer at 3500 rpm for 10 min to fully disperse BN-NH_2_ in deionized water. Subsequently, AE was added to the aqueous suspension of BN-NH_2_ and the mixture was homogenized at the same stirring speed for 5 min. After that, AE/BN-NH₂ films were prepared by tape casting the resulting slurry. During the tape-casting procedure, the films thickness were controlled between 50 and 70 μm, and the AE/BN-NH_2_ films were dried in an oven at 60 °C. In the next step, the prepared AE/BN-NH_2_ films were placed into a vacuum press for processing. The temperature of the machine was set at 160 °C, with a heating time of 100 s, a vacuum-holding time of 20 s and an applied pressure of 10 t. Finally, V-AE/BN-NH_2_ films can be obtained. For a comprehensive comparison, the preparation of V-AE/BN films were the same as that of V-AE/BN–NH_2_ film, except that the BN was unmodified.

***S1.4 Density functional theory simulation of the interface APTES and BN***

First-principles calculations were performed using the density functional theory (DFT) framework as implemented in the CASTEP package.[S1] The interactions between valence electrons and ionic cores were described using ultrasoft pseudopotentials. The exchange-correlation energy was treated within the generalized gradient approximation (GGA) using the Perdew-Burke-Ernzerhof (PBE) functional.[S2] A plane-wave basis set with a kinetic energy cutoff of 500 eV was employed, which was carefully tested to ensure convergence of total energies within 1×10^-6^ eV/atom. The Brillouin zone was sampled using a Monkhorst-Pack k-point grid of 2×2×1 for structural relaxation and static calculations. [S3] A vacuum layer of at least 20 Å was introduced along the out-of-plane direction to eliminate spurious interactions between periodic images.

The BN substrate was modeled using a periodic supercell, and all atomic positions were fully relaxed until the residual forces on each atom were less than 0.01 eV/Å, while the total energy convergence threshold was set to 1×10^-8^ eV. During geometry optimization, both the adsorbate molecules and the BN layers were allowed to relax unless otherwise specified. Long-range van der Waals interactions were considered using the DFT-D3 method of Grimme to accurately describe the weak interfacial interactions between the molecule and the BN substrate. [S4]

Following geometry optimization, static self-consistent calculations were performed to obtain accurate total energies for adsorption and binding energy analyses. The adsorption energy (*E*_abs_) was calculated according to:

$\text{E}_{\text{ads}}\text{=}\text{E}_{\text{APTES/BN}}\text{-}\text{E}_{\text{APTES}}\text{-}\text{E}_{\text{BN}}$ (S1)

where *E*_APTES/BN_ is the total energy of the molecule adsorbed on the BN, *E*_BN_ is the energy of the isolated BN substrate, and *E*_APTES_ is the energy of the isolated molecule calculated in the same supercell.

To analyze interfacial charge redistribution upon adsorption, the charge density difference (Δ*ρ*) was calculated as:

$\text{∆}\text{ρ}\text{=}\text{ρ}_{\text{APTES/BN}}\text{-}\text{ρ}_{\text{BN}}\text{-}\text{ρ}_{\text{APTES}}$ (S2)

where *ρ*_APTES/BN_, *ρ*_BN_, *ρ*_APTES_ represent the charge densities of the combined system, isolated BN, and isolated molecule, respectively, all evaluated using the same atomic positions as in the optimized adsorption configuration. The charge density difference was visualized using an isosurface value of 0.01 e/Å^3^. The charge density difference was calculated based on self-consistent charge densities obtained from CASTEP and visualized using the VESTA software. [S5]

***S1.5*** ***The analysis of orientation for the cast scraping using the Navier-Stokes equation.***


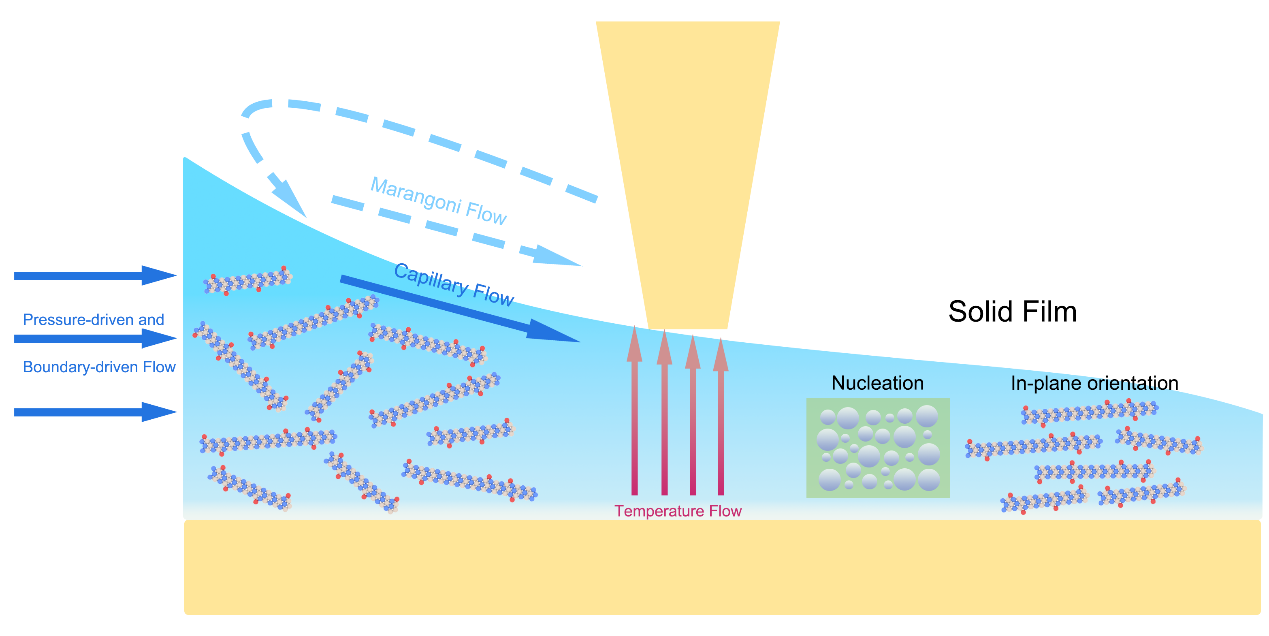


**Fig. T1** Detailed schematic of the cast scraping process.

Meniscus-guided coating (MGC) involves translating​ a meniscus across a substrate via a coating head or viscous forces, which guides and controls​ the film deposition process. In most MGC methods, the solution comes into contact with parts of the coating apparatus forming a meniscus at the liquid-air interface that bridges the coating head and the substrate. At this interface, the solution, substrate, and air meet to form a three-phase contact line. Before solution enters the region under the influence of the meniscus, the velocity field established by the coating head can itself alter the morphology of the resulting thin film. For simple blade coating with 0° tilt angle (blade parallel to the substrate), the system resembles classical parallel plate Couette flow, and the Navier-Stokes equation can be solved exactly to yield a linear velocity profile. When the gap distance between the two plates is very small, the Reynolds number

$\text{Re= }\frac{\text{ρν}\text{L}}{\text{μ}}$ (S3)

where *ρ* is the fluid density, *ν* is the relative velocity of the parallel plates, L is the gap distance, and *μ* is the fluid viscosity. In the evaporative regime at the advancing meniscus​ downstream of the coating head, the underlying​ processes of meniscus-guided coating are complex and interrelated. In the lubrication limit (Re ≪ 1) (see Supplementary Table S1.1 for specific parameters), the fluid velocity profile beneath the coating head is a superposition of a pressure-driven (parabolic) flow and a shear-driven (linear) flow due to boundary motion, as shown in **Fig. S**1. Subjected to the shear strain from the flow velocity gradient, the composites experience both uniaxial alignment in the coating direction and enhanced polymer aggregation and nucleation.[6] The actual results are shown in **Fig. S5**.

***S1.6*** ***The calculation of the index of Hermans orientation factor utilizing WXRD results***

Anisotropy vector:

$\text{S=}\left( \begin{matrix} \text{S}_{\text{11}} & \text{S}_{\text{12}} \\ \text{S}_{\text{21}} & \text{S}_{\text{22}} \end{matrix} \right)$ (S4)

These elements are defined as follows:

$\text{S}_{\text{11}}\text{=}\left\langle\text{cos}^{\text{2}}\left. \text{Ѱ} \right\rangle\right.\text{=}\frac{\int_{\text{0}}^{\text{2π}} \text{dѰ}\text{I(Ѱ)}\text{cos}^{\text{2}} \text{Ѱ}}{\int_{\text{0}}^{\text{2π}} \text{dѰ}\text{I(Ѱ)}}$ (S5)

$\text{S}_{\text{22}}\text{=}\left\langle\text{sin}^{\text{2}}\left. \text{Ѱ} \right\rangle\right.\text{=}\frac{\int_{\text{0}}^{\text{2π}} \text{dѰ}\text{I(Ѱ)}\text{sin}^{\text{2}} \text{Ѱ}}{\int_{\text{0}}^{\text{2π}} \text{dѰ}\text{I(Ѱ)}}$ (S6)

$\text{S}_{\text{12}}\text{=}\text{S}_{\text{21}}\text{=}\left\langle\text{sin}\left. \text{Ѱ*}\text{cosѰ} \right\rangle\right.\text{=}\frac{\int_{\text{0}}^{\text{2π}} \text{dѰ}\text{I}\left( \text{Ѱ} \right)\text{*}\text{sin}\text{Ѱ}\text{*}\text{cosѰ}}{\int_{\text{0}}^{\text{2π}} \text{dѰ}\text{I(Ѱ)}}$ (S7)

Here, Ψ denotes the azimuthal angle in the imaging convention, defined as increasing counterclockwise from the positive X-axis (the 3 o’clock position). Notably, the trace of the matrix S​ is 1, a property that is invariant under rotation.

Matrix (S4) is diagonalizable by a similarity transformation. Its eigenvalues are given by

$\text{λ}_{\text{1,2}}\text{=}\frac{\text{1}}{\text{2}}\left( \text{S}_{\text{11}}\text{+}\left. \text{S}_{\text{22}} \right) \right.\text{±}\sqrt{\left( \text{S}_{\text{11}}\text{-}\left. \text{S}_{\text{22}} \right) \right.^{\text{2}}\text{+4}\text{S}_{\text{12}}^{\text{2}}}$ (S8)

The diagonalization can be expressed as

$\text{T}^{\text{-1}}\text{ST=}\left( \begin{matrix} \text{λ}_{\text{1}} & \text{0} \\ \text{0} & \text{λ}_{\text{2}} \end{matrix} \right)$ (S9)

where the transformation matrix represents a counterclockwise rotation by an angle x:

$\text{T=}\left( \begin{matrix} \cos\text{x} & \text{-}\sin\text{x} \\ \sin\text{x} & \cos\text{x} \end{matrix} \right)$ (S10)

Substituting the explicit eigenvalues (S8) into equation (S9) leads to the condition that the off-diagonal elements vanish when x satisfies

$\tan\text{2x}\text{=}\frac{\text{2}\text{S}_{\text{12}}}{\text{S}_{\text{11}}\text{-}\text{S}_{\text{22}}}$ (S11)

The angle x obtained from equation (S11) is the principal orientation angle which is the rotation required to align the coordinate system with the sample's intrinsic axes.

It is important to note that equation (S11) alone does not uniquely determine the quadrant of x, since both x and x+π/2 yield the same value of tan(2x). In practice, the correct quadrant is determined by analyzing the contextual data associated with the two equivalent angle candidates.

For an isotropic sample, the eigenvalues are equal λ₁ = λ₂. In this case, the similarity transformation in equation (S9) yields the identity matrix, and the orientation angle x becomes undefined because any rotation satisfies (S9) The degree of orientation is therefore quantified by the difference between the two eigenvalues (by definition λ₁ ≥ λ₂)

$\text{∆}\text{S=}\text{λ}_{\text{1}}\text{-}\text{λ}_{\text{2}}\text{=}\sqrt{{\text{(}\text{S}_{\text{11}}\text{-}\text{S}_{\text{12}}\text{)}}^{\text{2}}\text{+4}{\text{S}_{\text{12}}}^{\text{2}}}$ (S12)

While equation (S12) applies to low symmetries (e.g., single or double peaks in the azimuthal profile), the definition in (S7) must be adapted for four-fold symmetries (peaks at angles separated by π/2). Specifically, the transformation Ψ→2Ψ is required. However, that the original definition (S7) must still be used when calculating the orientation angle via equation (S11).

The Herman factor is defined as:

$\text{H=}\frac{\text{3}}{\text{2}}\frac{\int_{\text{0}}^{\frac{\text{π}}{\text{2}}} \text{dѰ}\sin\text{ѰI(Ѱ)}\text{cos}^{\text{2}} \text{Ѱ}}{\int_{\text{0}}^{\frac{\text{π}}{\text{2}}} \text{dѰ}\sin\text{ѰI(Ѱ)}}\text{-}\frac{\text{1}}{\text{2}}$ (S13)

where a value of H=1 denotes ideal​ face-on orientation (Ψ=0), H=-0.5 denotes ideal​ edge-on orientation (Ψ=π/2), and H= 0 denotes a completely random, isotropic structure. There is no azimuthal modulation in the data, we expect H to be 1. It is important to note that if data for the entire azimuthal range from 0 to π/2 are unavailable, then the calculation result is meaningless.[7]

***S1.7*** *Angle-resolved polarization Raman measurements of BN in films*


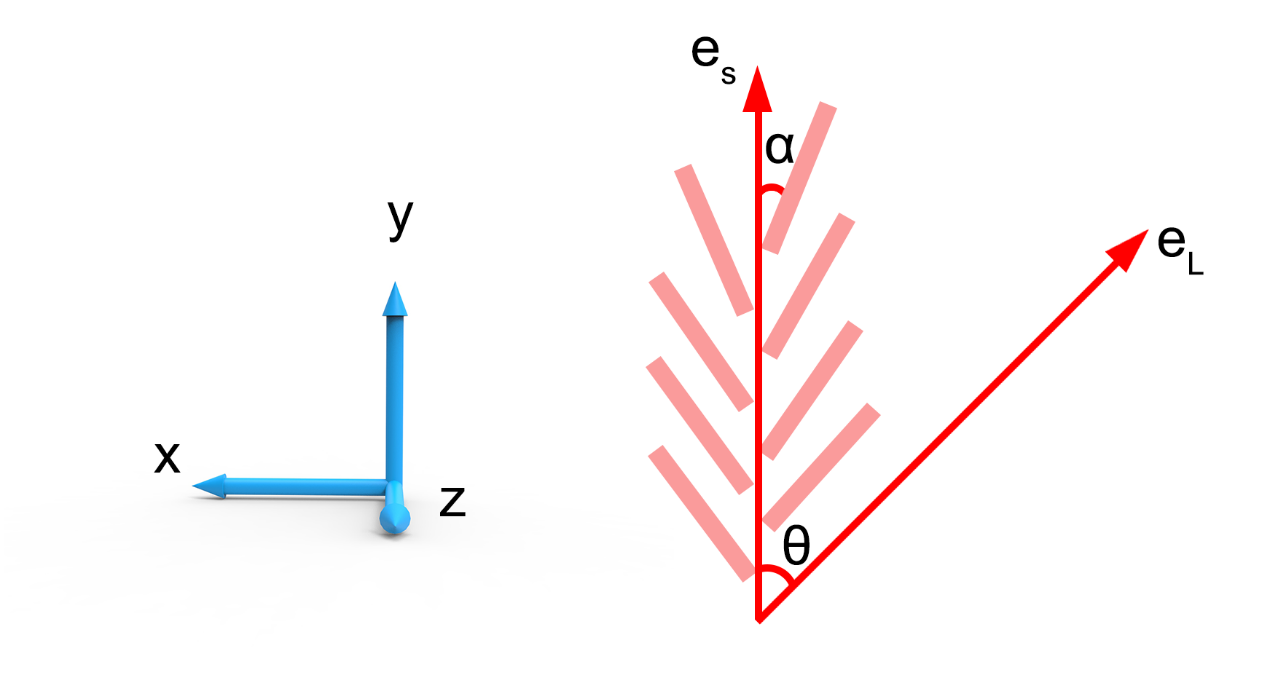


**Fig. T2** Schematic diagram of BN sheets alignment and the angle-resolved polarized Raman configuration.

Figure T2​ illustrates a simplified model of BN alignment and the configuration​ for angle-resolved polarized Raman measurements. The laboratory coordinate system (x, y, z) is represented by blue vectors. The x-axis is perpendicular, and the y-axis is parallel, to the basal plane of the film.​ The laser propagates along the z-direction, and its polarization direction​ makes an angle θ with the basal plane. The BN were assumed to be uniformly oriented, with their average alignment making an angle α relative to the y-aligned basal plane. The unit electric field vectors for the incident laser and the scattered Raman signal are defined as​ e_L_ and e_S_. The angle θ between the laser polarization and the basal plane​ was controlled​ by a half-wave plate in steps of​ 7.5°. Measurements were performed at θ=90° and 180° in addition to​ other angles. Accordingly, the​ cumulative Raman intensity of the G mode from all BN ​are calculated using the following equation [S8]:

$\text{I}_{\text{G}}\left( \text{θ} \right)\text{=}\frac{\text{1}}{\text{2}}\text{c}^{\text{2}}\text{cos}^{\text{2}} \text{α}\text{\{2+}\cos\left[ \text{2}\left( \text{α-θ} \right) \right]\text{+}\cos\text{[2(α+θ)]}\text{\}}$ (S14)

The intensity of the generated signal (I_G_) reaches its maximum and minimum values when the electric field vector of the incident polarized laser beam (e_L_) is parallel (θ=0° and 180°, corresponding to I_G_(‖)) or perpendicular (θ=90° and 270°), corresponding to I_G_(⊥) to the basal plane of the V-AE/BN-NH_2_ film, respectively. As shown in Fig. T2, the ratio I_G_(‖)/I_G_(⊥) is given by cot^2^α, where α denotes the alignment angle between the average orientation of the BN platelets and the film's length direction (y-axis). Specifically, this ratio increases monotonically with a decrease in α.

***S1.8*** ***Molecular dynamics simulation of the Micro-nano interface heat conduction efficiency***

All molecular dynamics simulations were performed using the Large-scale Atomic/Molecular Massively Parallel Simulator (LAMMPS, 2024 version).[S9] The polymer matrix was modeled using a COMPASS-type Class II force field, in which bonded interactions, including bond stretching, angle bending, dihedral torsion, and improper terms, were described by anharmonic Class II potentials with cross-coupling terms. During the polymer structure construction and initial equilibration stage, non-bonded interactions were described using a truncated 9-6 Lennard-Jones potential combined with short-range Coulombic interactions (cut) to improve computational efficiency. For subsequent thermal transport simulations, long-range Coulombic interactions were explicitly considered using the long formulation with the PPPM solver to ensure accurate energy conservation and heat flux evaluation. The monolayer BN was described using the Tersoff potential to accurately capture its covalent bonding characteristics. A hybrid potential scheme implemented in LAMMPS was employed to account for the different interaction types in the polymer/BN composite system.

The interfacial interactions between the polymer and BN were described using the same Class II force field to ensure a consistent and physically reasonable description of van der Waals interactions at the interface. Prior to thermal transport simulations, the system was fully equilibrated for 1,000,000 fs at a temperature of 350 K under the canonical (NVT) ensemble. During the equilibration process, atoms within 20 Å at the top and bottom of the simulation domain were fixed to suppress global translation and maintain structural stability.

Non-equilibrium molecular dynamics (NEMD) simulations were subsequently conducted to investigate thermal transport. Two regions with a thickness of 10 Å adjacent to the fixed layers were defined as the heat source and heat sink, respectively. A constant heating and cooling power of 0.1 (in LAMMPS real units) was applied to the corresponding regions, while the remaining atoms were evolved under the microcanonical (NVE) ensemble. The system was first evolved for 1,000,000 fs to establish a steady-state temperature gradient. Subsequently, an additional 1,000,000 fs were performed, during which the instantaneous temperature was recorded at every time step. The final temperature profile was obtained by time-averaging the collected data to minimize statistical fluctuations. The temperature gradient was extracted from the linear region of the steady-state temperature profile, excluding the heat source and sink regions.


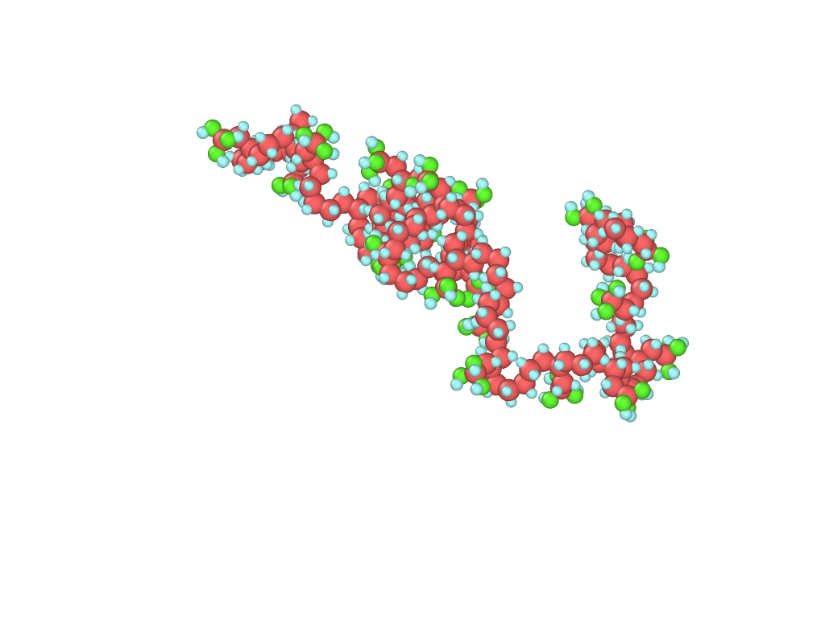

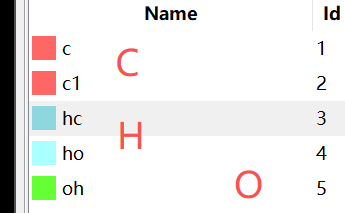


**Fig. T3** The details of the Non-equilibrium molecular dynamics simulations.

***S1.9 The analysis of the thermal conductivity for the V-AE/BN-NH_2_ films using the metal foam theory.***

The thermal conductivity of the composite (*K*_composite_) containing an interconnected BN framework can be predicted as a function of the volume fraction (*V*) using the metal foam theory described by Equation (S15). [S10]

$\text{K}_{\text{composite}}\text{=}\text{K}_{\text{framework}}\text{+}\left（ \text{1-}\text{V}_{\text{BN}} \right）\text{K}_{\text{AE}}$ (S15)

where *V*_BN_ is the volume fraction of BN; *K*_AE_ is the thermal conductivity of the polymer matrix; and *K*_framework_ is the effective thermal conductivity contributed by the BN strut, calculated using Equation (S16). [S11]

$\text{K}_{\text{framework}}\text{=}\left\langle\text{cos}^{\text{2}} \text{θ} \right\rangle\text{V}_{\text{BN}}\text{K}_{\text{strut}}$ (S16)

Where *K*_strut_is the thermal conductivity of a single BN framework; θ is the angle between the framework orientation and the heat flow direction; and the angle bracket indicates denotes the average over all struts.

When BN is randomly oriented, it forms an isotropic framework. For such a system, the orientation average satisfies ⟨cos^2^θ⟩ = 1/3, implying that the thermal conductivity enhancement is equal in all three directions (x, y, and z axes). Therefore, the thermal conductivity of the embedded framework can be estimated using Equation (S17).[S12]

$\text{K}_{\text{∥}}\text{≈}\text{K}_{\text{⊥}}\text{=}\frac{\text{1}}{\text{3}}\text{V}_{\text{BN}}\text{K}_{\text{strut}}\text{+1-}\text{V}_{\text{AE}}\text{K}_{\text{AE}}$ (S17)

where *K*_∥_ is​ the in-plane thermal conductivity of the V-AE/BN-NH₂ film, with a typical value of​ approximately 17 W m^-1^ K^-1^; *K*_AE_ is the bulk thermal conductivity of the acrylic ester (0.19 W m^-1^ K^-1^); *V*_BN_ is the volume fraction of V-AE/BN-NH_2_ in the composite (≈ 28.8 vol%), based on the TGA analysis. Therefore, the solid thermal conductivity of an individual BN strut (*K*_strut_) for the V-AE/BN-NH_2_ obtained from Eq. (S15) is 174 W m^-1^ K^-1^.

When BN is perfectly oriented, it constitutes an anisotropic framework, where the orientational average satisfies ⟨cos^2^θ⟩ = 1. Consequently, the thermal conductivity of the embedded framework can be estimated using Equation (S18).

$\text{K}_{\text{∥}}\text{=}\text{V}_{\text{BN}}\text{K}_{\text{strut}}\text{+1-}\text{V}_{\text{AE}}\text{K}_{\text{AE}}$ (S18)

where *K*_∥_ is​ a value of​ approximately 78 W m^-1^ K^-1^, *K*_AE_ is 0.19 W m^-1^ K^-1^, *V*_BN_ is 68 vol%. Therefore, the solid thermal conductivity of an individual BN strut (*K*_strut_) for the V-AE/BN-NH_2_ obtained from Equation (S18) is 114 W m^-1^ K^-1^.

Based on the calculation, the thermal conductivity of the BN framework​ in the composite is estimated to be 120 W m^-1^ K^-1^. According to Equation (S19) and equation (S20), Orientation factor (f) can be calculated:

$\text{K}_{\text{composite}}\text{=}\text{V}_{\text{BN}}\text{K}_{\text{strut}}\text{ }\left\langle\text{cos}^{\text{2}} \text{θ} \right\rangle\text{+}\text{V}_{\text{AE}}\text{K}_{\text{AE}}\text{+}\text{V}_{\text{air}}\text{K}_{\text{air}}$ (S19)

$\text{f=}\frac{\text{3}\text{cos}^{\text{2}} \text{θ}\text{-1}}{\text{2}}$ (S20)

Where *K*_composite_ is the thermal conductivity of V-AE/BN-NH_2_ film, θ is the angle between the BN struts and the heat conduction direction, and ⟨cos^2^θ⟩ denotes the average over all struts. *V*_BN_, *V*_AE_ and *V*_air_ denote the volume fractions of BN, the acrylic matrix and the entrapped air, respectively. Correspondingly, *K*_strut_, *K*_AE_, and *K*_air_ are the thermal conductivities of a single BN strut (120 W m^-1^ K^-1^), the acrylic matrix (0.19 W m^-1^ K^-1^), and air (0.026 W m^-1^ K^-1^). See Supplementary Table S3.4 for details of the specific parameters.

**S2 Supplementary Figures**

**
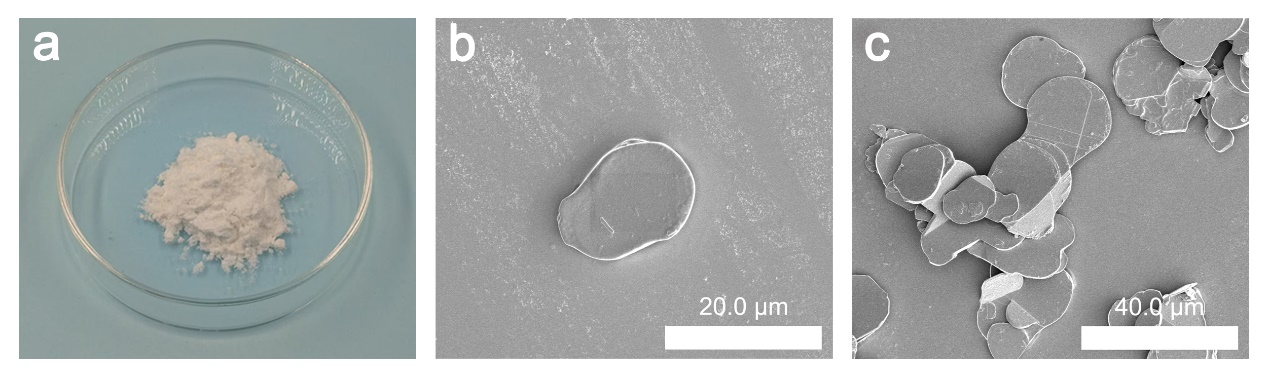
**

Fig. S1 The optical diagram and SEM image of pristine BN powder. (a) The optical diagram of BN powder. (b, c) The SEM image of BN powder.


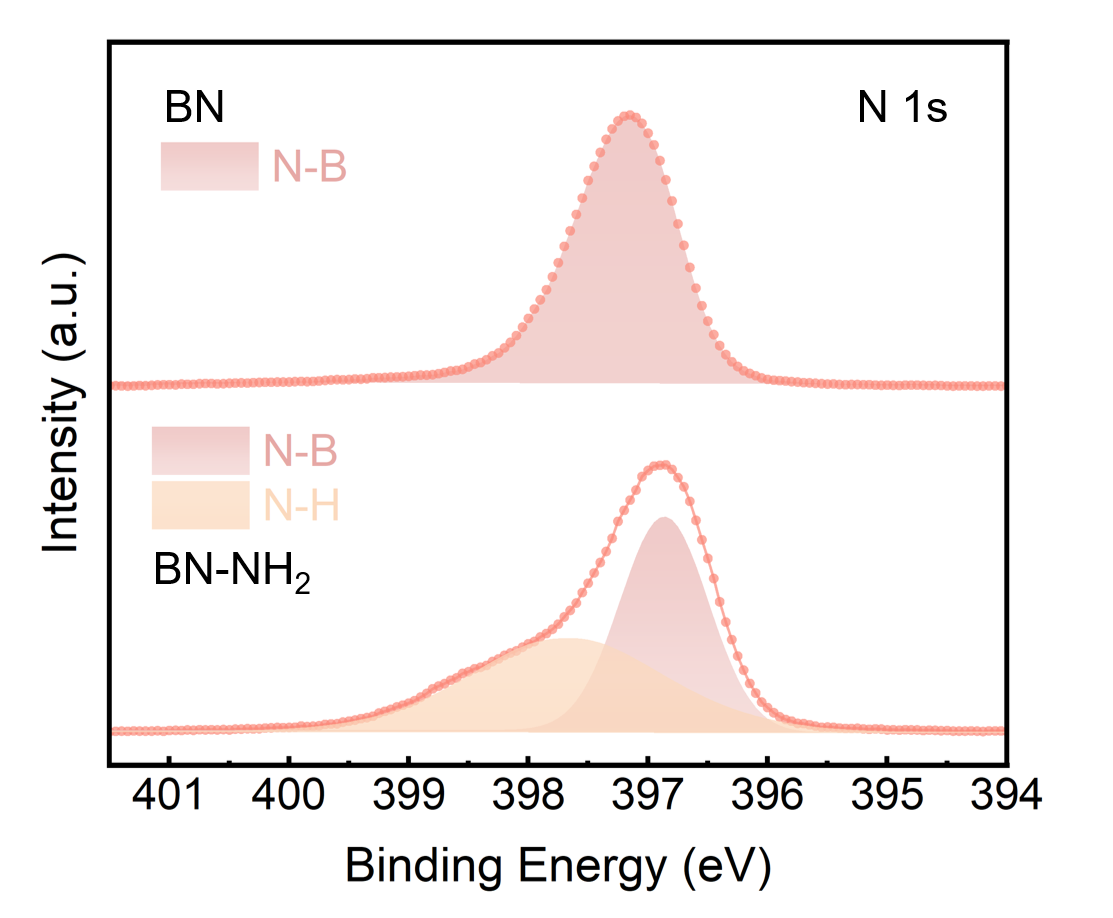


**Fig. S2** The XPS analysis of N 1s spectra of BN and BN-NH_2_.

**
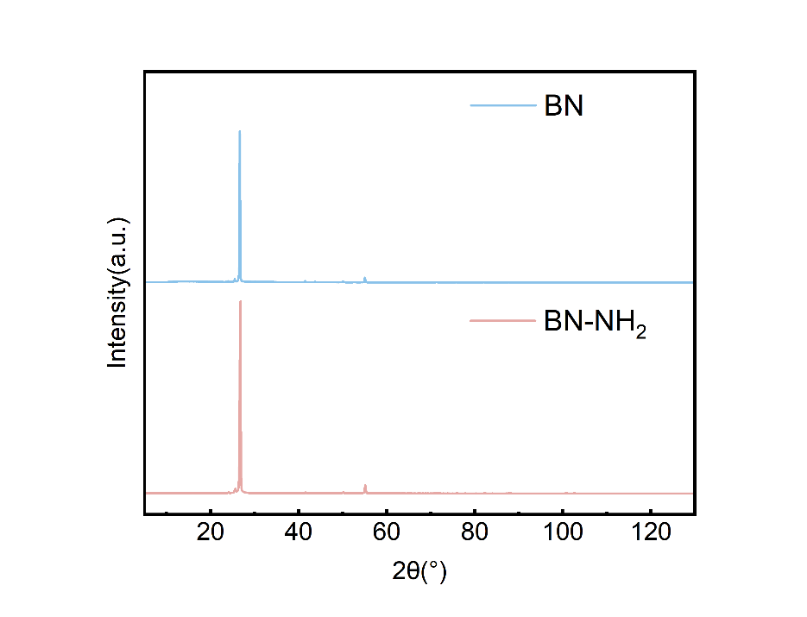
**

Fig. S3 The XRD patterns of BN and BN-NH_2_.


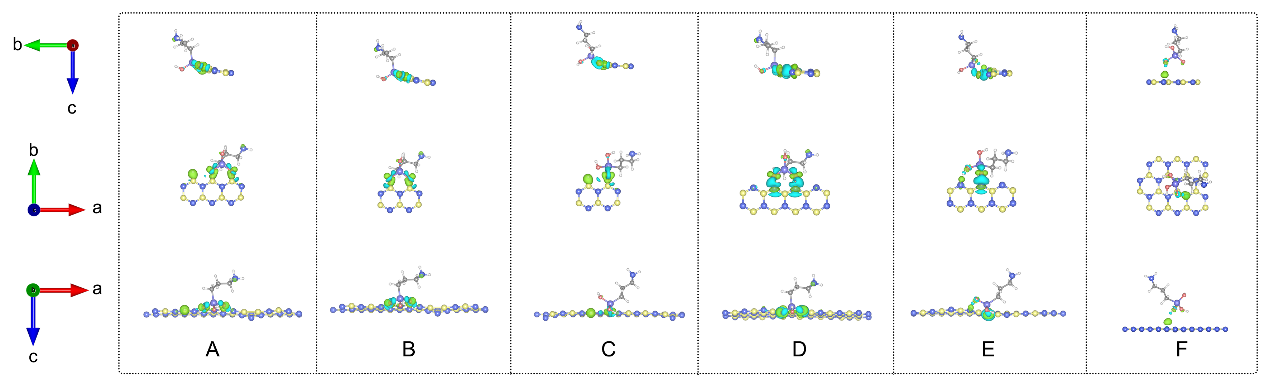


Fig. S4 The binding energy of APTES at different BN sites by DFT calculation. Green and cyan regions represent electron accumulation and depletion, respectively.


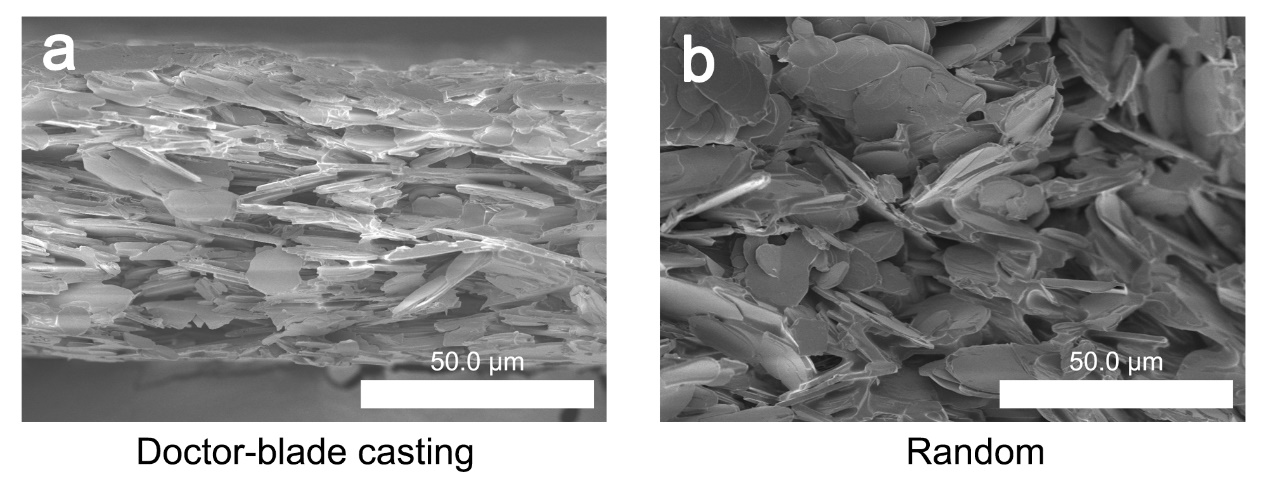


Fig. S5 The effect of cast scraping on films orientation. (a) The SEM image of the film fabricated by doctor-blade casting. (b)The SEM image of the film fabricated by gravity.


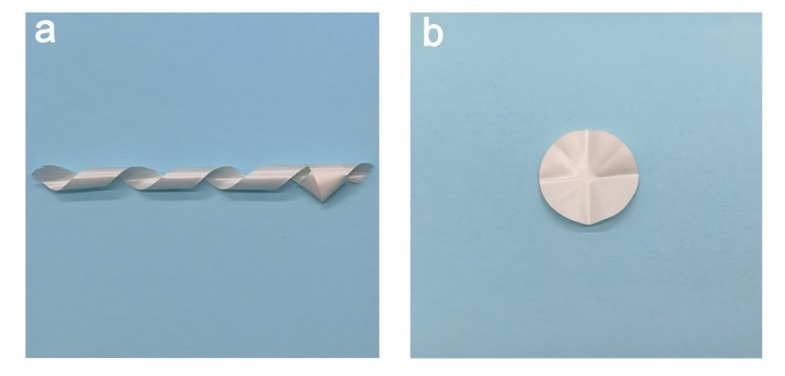


Fig. S6 (a) The flexibility of the film. (b) The foldability of the film.


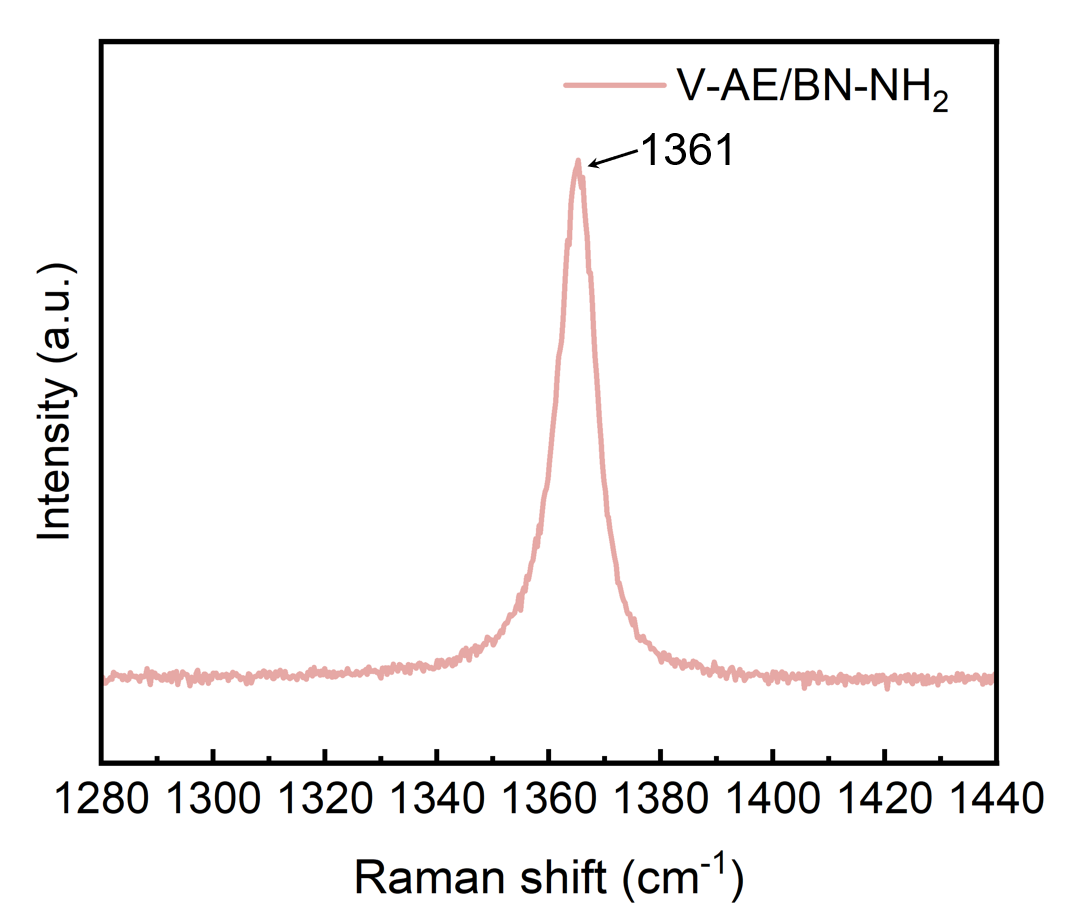


Fig. S7 The Raman spectra of the V-AE/BN-NH_2_ film.


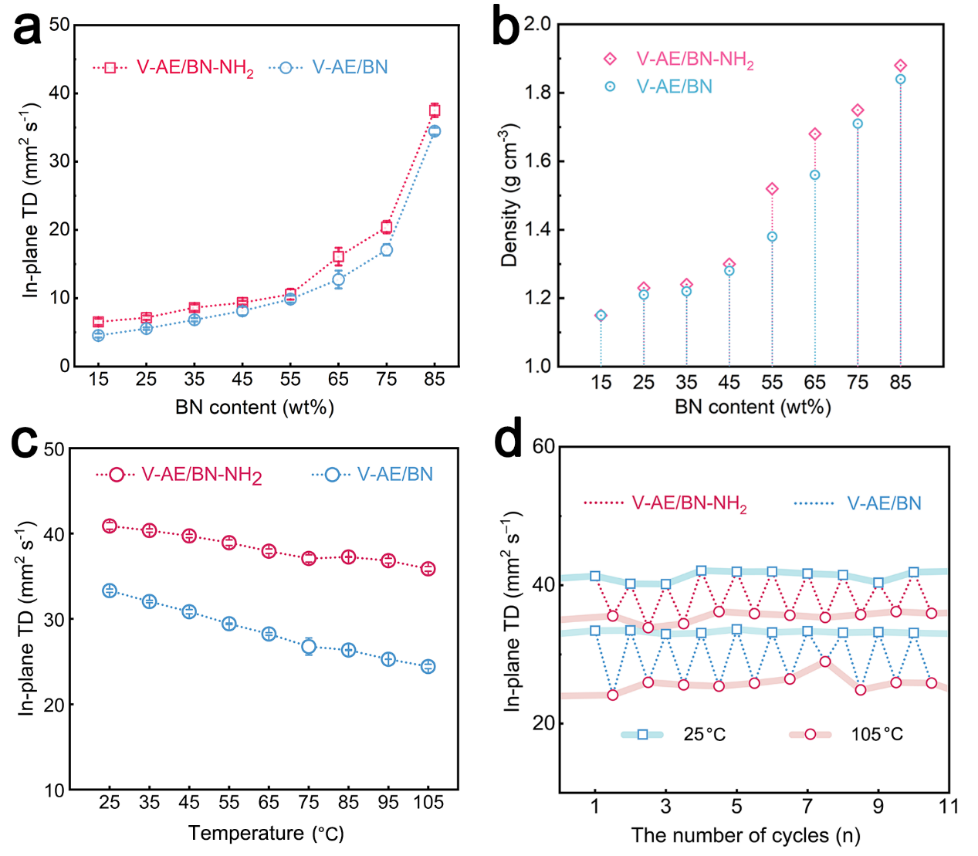


Fig. S8 (a)Thermal diffusivity of V-AE/BN-NH_2_ and V-AE/BN films with the different BN content. (b) Density of V-AE/BN-NH_2_ and V-AE/BN films with different BN content. (c) Thermal diffusivity of V-AE/BN-NH_2_ and V-AE/BN films at the different temperatures. (d) Thermal diffusivity of V-AE/BN-NH_2_ and V-AE/BN films over ten thermal cycles.


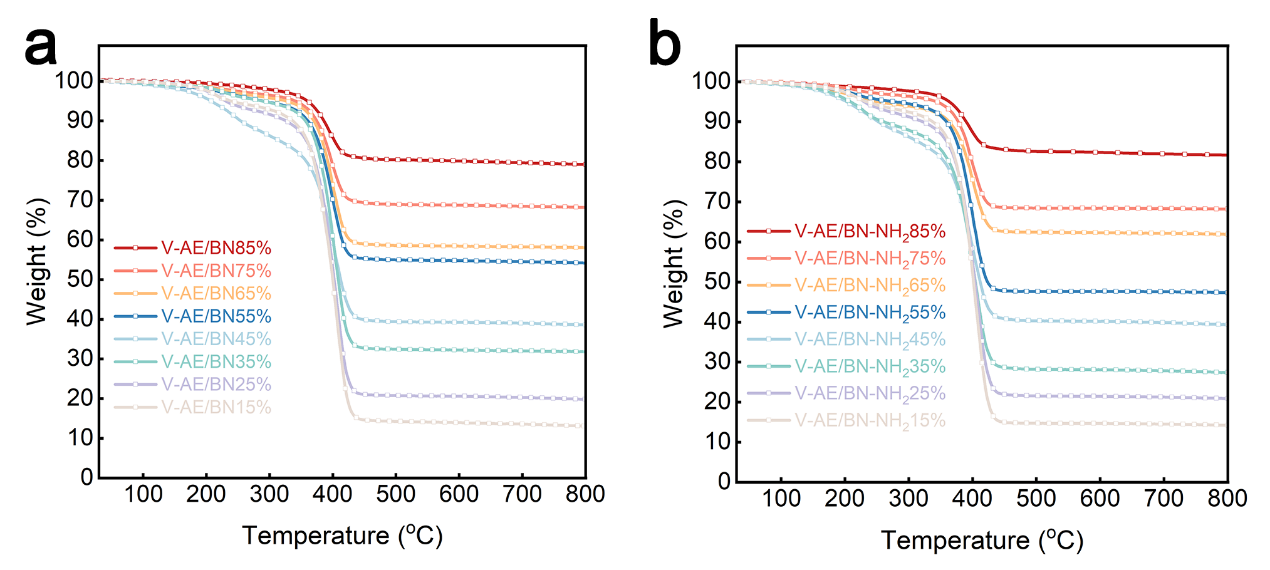


Fig. S9 TGA curves of V-AE/BN and V-AE/BN-NH_2_ films in the nitrogen atmosphere. (a) The TGA curves of V-AE/BN film. (b) The TGA curves of V-AE/BN-NH_2_ film.

Based on the TGA curves of V-AE/BN and V-AE/BN-NH_2_ films, the volume fraction of the films can be calculated by equation (S21):

$\text{V}_{\text{BN}}\text{=}\frac{\frac{\text{ω}_{\text{BN}}}{\text{ρ}_{\text{BN}}}}{\frac{\text{1}}{\text{ρ}_{\text{composite}}}}$ (S21)

Where *ω*_BN_ is the mass fraction of BN, *ρ*_BN_ is the density of BN, and *ρ*_composite_ is the density of film.

**
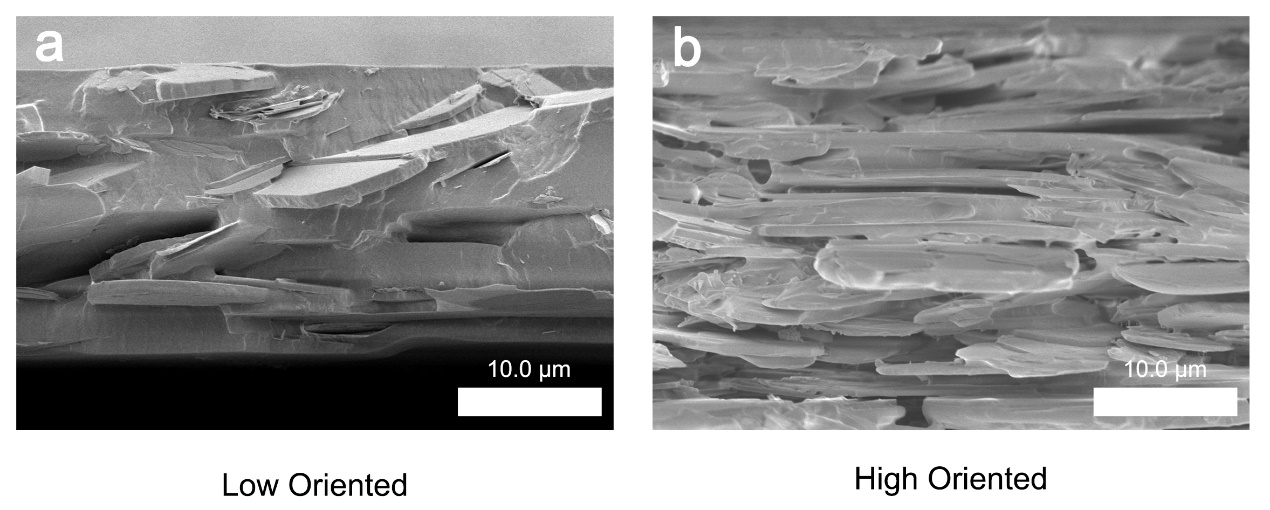
**

Fig. S10 The SEM image of the films at varied orientations. (a) The SEM image of the low oriented film. (b) The SEM image of the high oriented film.


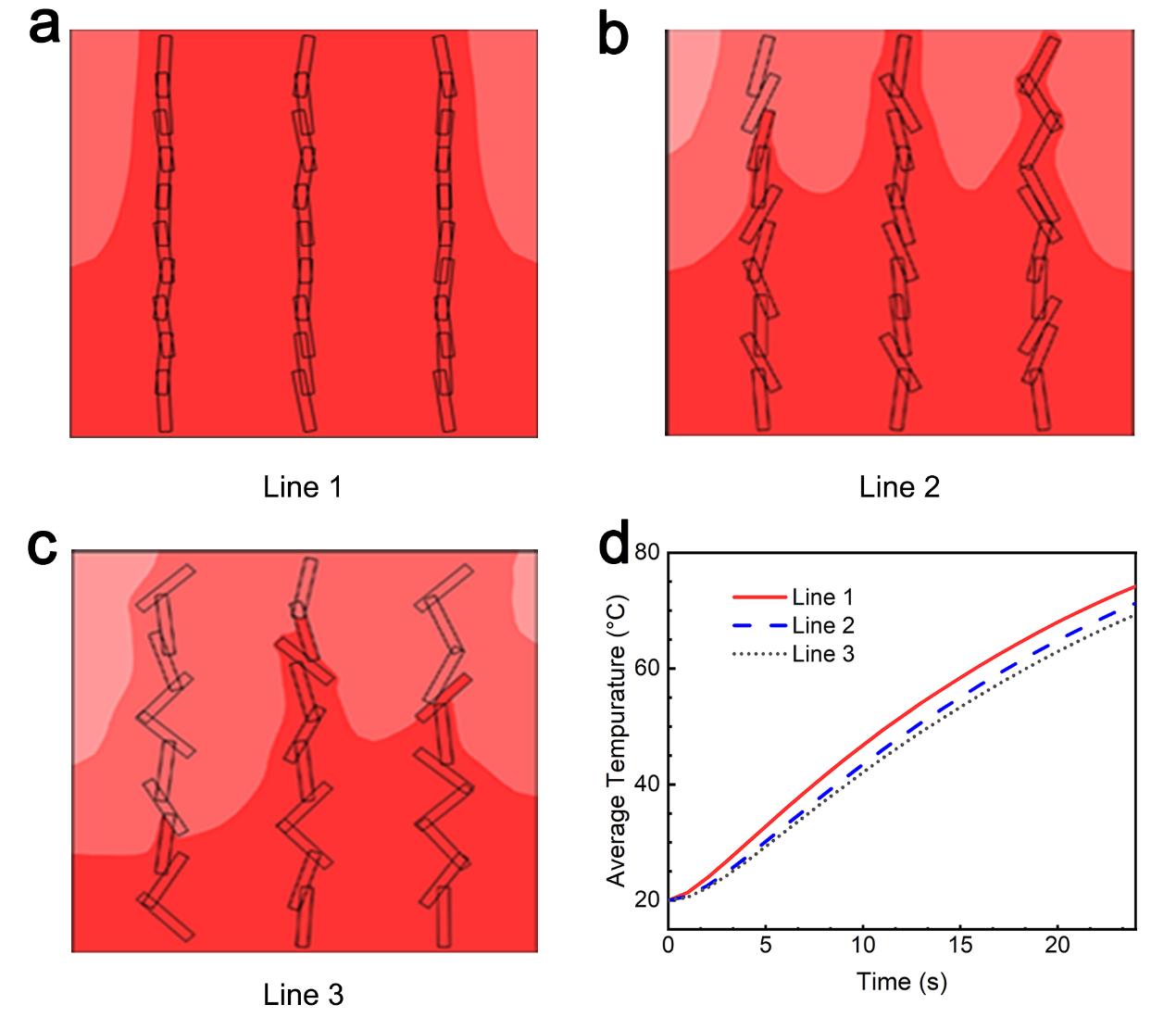


Fig. S11 The average temperature curves at the top position calculated by three different simulation models. (a-c) Three different simulation models. (d) The calculated results of the average temperature at the top position.


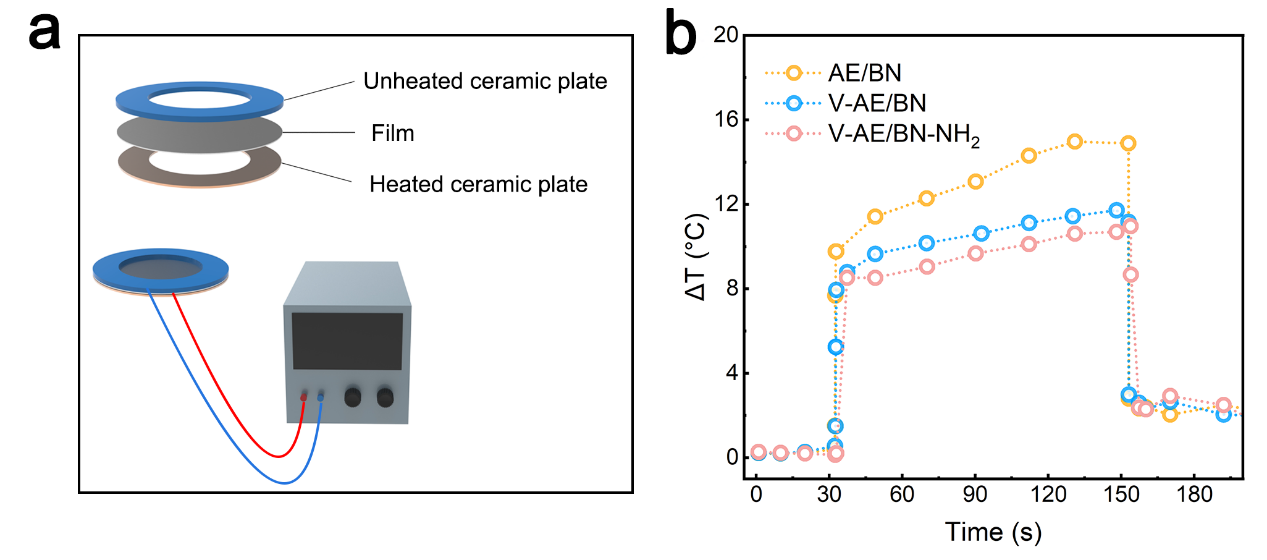


Fig. S12 Schematic of the device and the measured temperature difference curve. (a) The schematic configuration of the uniform heat performance test system. (b) The results of uniform heat performance test.


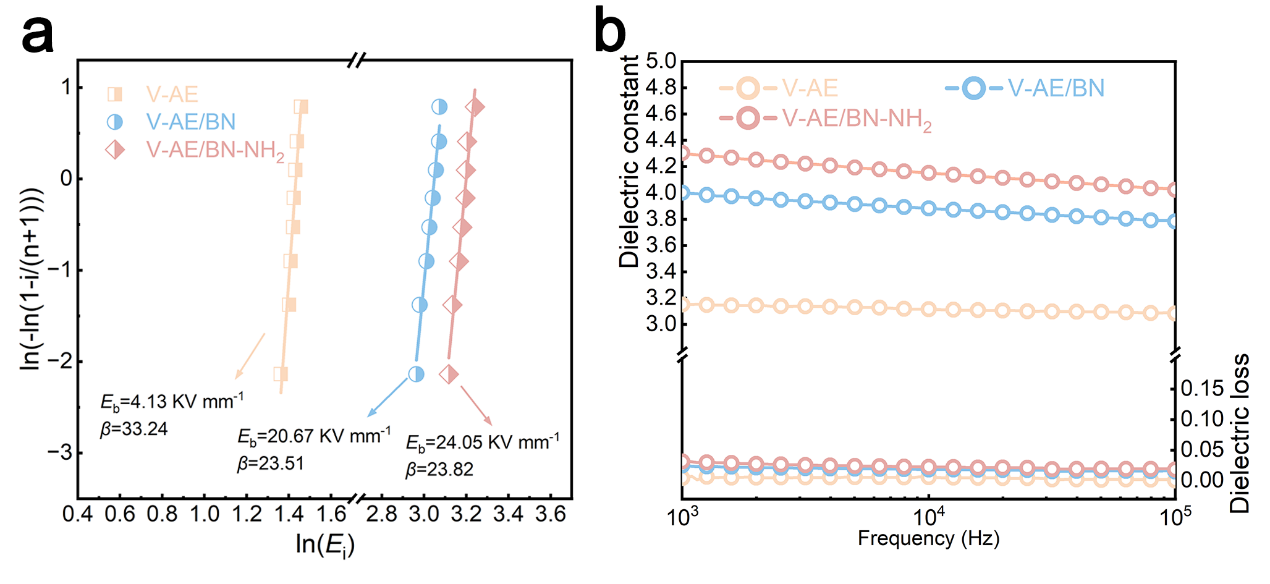


Fig. S13 The breakdown strength, dielectric constant and dielectric loss of the films. (a) The breakdown strength (DC) of the films. (b) The dielectric constant and dielectric loss of the films.


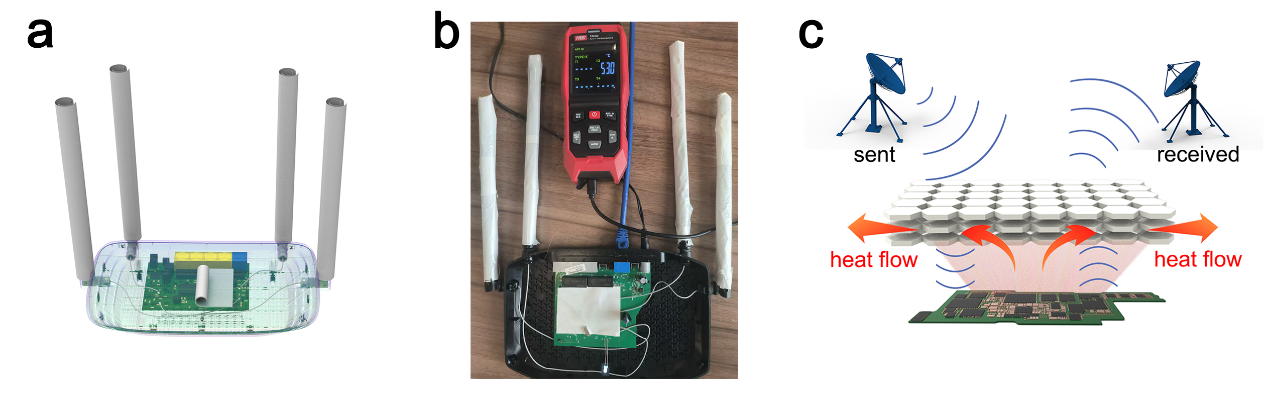


Fig. S14 Schematic diagram of the actual test device for wave transparency.

As shown in **Fig. S**14, a router-based setup was constructed to test the wave transparency of V-AE/BN-NH_2_ film. Under the spatial constraints from the transceiver and heating element, V-AE/BN-NH_2_ film was covered to the antenna and circuit board, and the signal stability of the router was subsequently evaluated.


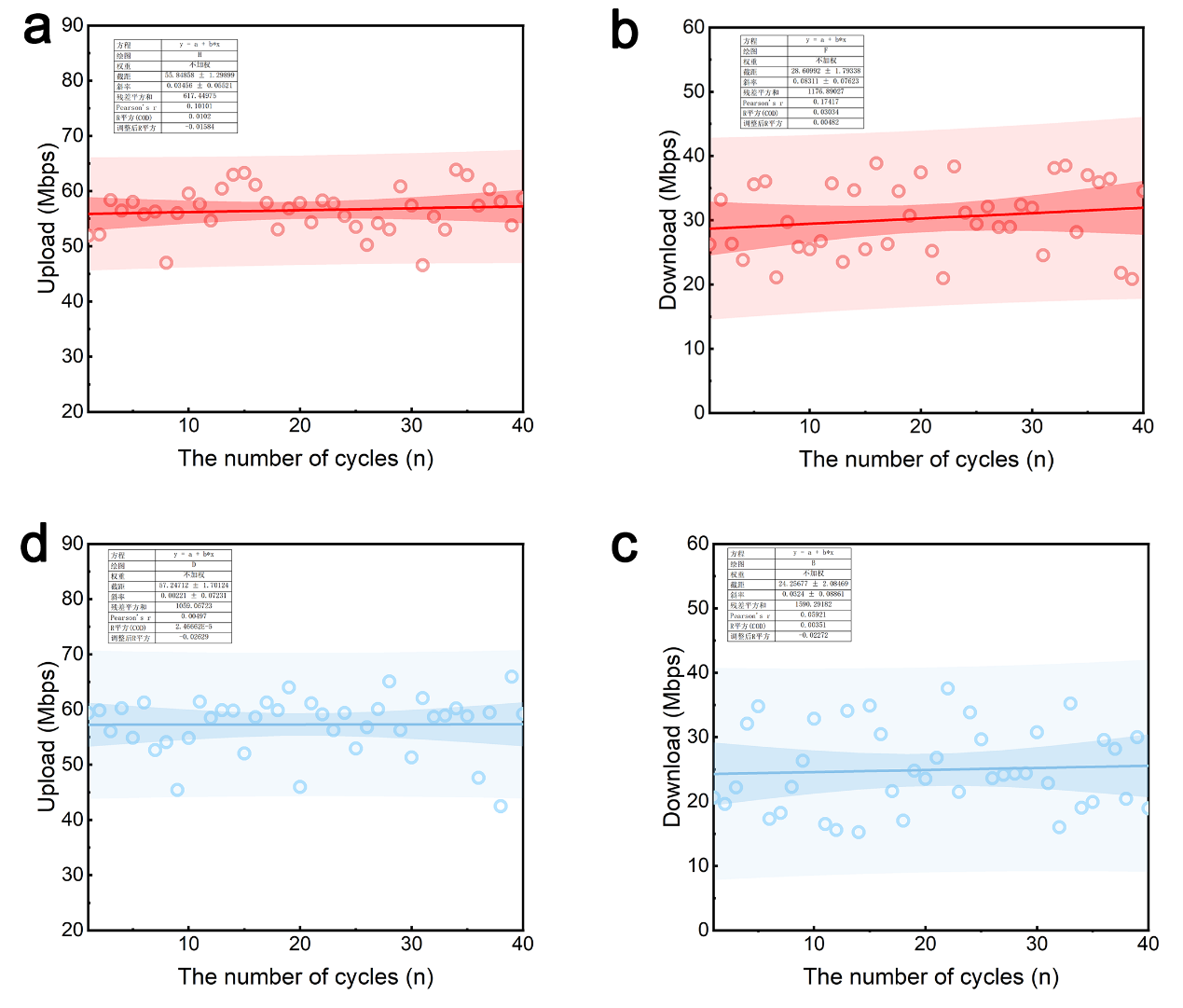


Fig. S15 The trends in actual web speed test result. (a, b) The trends in upload and download speed of the film-coated router. (c, d) The trends in upload and download speed of the exposed router.

Figure S15 presents the 98% confidence interval derived from 40 network tests. Obviously, V-AE/BN-NH_2_ film-covered router (red curve) is significantly more stable than the exposed router (blue curve).

**S3 Supplementary Tables**

*S3.1* ***The calculated parameter of the Navier-Stokes equation***

| Re | *ρ* (g cm^-3^) | *ν* (mm s^-1^) | L (mm) | *μ* (mPa s) |
| --- | --- | --- | --- | --- |
| 0.0017 | 1.2 | 15.625 | 0.48 | 5375 |
|  |  |  |  | 5396 |
|  |  |  |  | 5355 |

***S3.2 The parameters for the calculation of in-plane thermal conductivities of acrylic ester and the V-AE/BN and V-AE/BN-NH_2_ films***

The specific heat capacity (*C*_p_) of all samples was evaluated using a differential scanning calorimeter (DSC) analysis.

| Sample | Boron Nitride content  (wt %) | Thermal diffusivity (mm^2^ s^-1^) | Specific heat capacity  (J g^-1^ K^-1^) | Density  (g cm^-3^) | Thermal conductivity (W m^-1^ K^-1^) |
| --- | --- | --- | --- | --- | --- |
| Pure AE | 0 | 0.09 | ≈1.98 | ≈1.06 | 0.19 |
| V-AE/BN | 15 | 4.54±0.29 | ≈1.59 | ≈1.15 | 8.30±0.53 |
|  | 25 | 5.55±0.17 | ≈1.55 | ≈1.21 | 10.41±0.32 |
|  | 35 | 6.84±0.24 | ≈1.44 | ≈1.22 | 12.01±0.43 |
|  | 45 | 6.88±0.83 | ≈1.42 | ≈1.28 | 12.02±1.50 |
|  | 55 | 8.92±0.36 | ≈1.18 | ≈1.38 | 14.52±0.58 |
|  | 65 | 12.74±1.30 | ≈1.12 | ≈1.56 | 22.25±2.27 |
|  | 75 | 17.08±0.84 | ≈1.04 | ≈1.71 | 30.37±1.50 |
|  | 85 | 34.47±0.51 | ≈0.98 | ≈1.84 | 62.15±0.92 |
| V-AE/BN-NH_2_ | 15 | 6.54±0.46 | ≈1.56 | ≈1.15 | 11.73±0.83 |
|  | 25 | 7.14±0.33 | ≈1.54 | ≈1.23 | 13.53±0.63 |
|  | 35 | 8.62±0.47 | ≈1.4 | ≈1.24 | 14.96±0.82 |
|  | 45 | 9.35±0.27 | ≈1.38 | ≈1.30 | 16.78±0.47 |
|  | 55 | 10.57±0.78 | ≈1.28 | ≈1.52 | 20.56±1.53 |
|  | 65 | 14.05±1.20 | ≈1.21 | ≈1.68 | 28.57±2.44 |
|  | 75 | 23.74±0.89 | ≈1.03 | ≈1.75 | 42.79±1.60 |
|  | 85 | 41.76±0.74 | ≈1 | ≈1.88 | 78.50±1.39 |

*S3.3* ***Comparison of in-plane thermal conductivity between our V-AE/BN-NH_2_ film with thermally conductive insulating films reported in the literature***

| TC of Matrix  (W m^-1^ K^-1^) | TC of composite  (W m^-1^ K^-1^) | Loading | TCE | Method | Ref. |
| --- | --- | --- | --- | --- | --- |
| 0.16 | 6.51 | 20 wt% | 39.69 | electrospinning | [S13] |
| 0.23 | 24.4 | 65 wt% | 105.09 |  | [S14] |
| 0.18 | 18.76 | 35 wt% | 103.22 |  | [S15] |
| 0.47 | 6.99 | 10 wt% | 13.78 |  | [S16] |
| 0.24 | 4.09 | 57.6 wt% | 16.04 |  | [S17] |
| 1.4 | 10.7 | 60 wt% | 6.64 | vacuum-assisted filtration | [S18] |
| 3.45 | 11.51 | 40 wt% | 2.34 |  | [S19] |
| 1.6 | 40.25 | 90 wt% | 24.16 |  | [S20] |
| 1.68 | 4.74 | 90 wt% | 1.82 |  | [S21] |
| 4.4 | 21.08 | 50 wt% | 3.79 | solvent exchange | [S22] |
| 2.3 | 5.3 | 50 wt% | 1.30 |  | [S23] |
| 6.74 | 45.5 | 40 wt% | 5.75 |  | [S24] |
| 1.7 | 14.1 | 30 wt% | 7.29 |  | [S25] |
| 0.2 | 12.7 | 10 wt% | 62.50 | Tape-casting | [S26] |
| 3.57 | 4.44 | 24.4 wt% | 0.24 |  | [S27] |
| 0.19 | 73.4 | 85 wt% | 329.26 |  | [S28] |
| 0.19 | 78.5 | 85 wt% | 412.16 |  | This work |

***S3.4 The key parameters for calculating the 〈cos^2^θ〉 and orientation parameter (f)***

*V*_BN_ is obtained based on the TGA analysis shown in **Fig. S**9. *V*_air_ is the volume fraction of the existing air bubbles in the composites, and can be calculated using the equation of *V*_air_ = 1 - *ρ*_composite_/*ρ*_theoretical_, where *ρ*_composite_ and *ρ*_theoretical_ are the measured and theoretical density of the boron nitride framework/acrylic ester composites, respectively. *V*_AE_ is obtained based on the equation of *V*_AE_ = 1 - *V*_BN_ - *V*_air_.

| Boron Nitride  Content (wt%) | *ρ*_composite_ (g cm^-3^) | *ρ*_theoretical_ (g cm^-3^) | *V*_BN_ | *V*_air_ | *V*_AE_ | *K*_strut_  (W m^-1^ K^-1^) | *K*_AE_  (W m^-1^ K^-1^) | *K*_air_  (W m^-1^ K^-1^) |
| --- | --- | --- | --- | --- | --- | --- | --- | --- |
| 47.03 | 1.38 | 1.71 | 0.29 | 0.19 | 0.52 | 120 | 0.19 | 0.026 |
| 61.53 | 1.56 | 1.83 | 0.43 | 0.15 | 0.43 |  |  |  |
| 68.11 | 1.71 | 1.95 | 0.52 | 0.12 | 0.36 |  |  |  |
| 81.61 | 1.88 | 2.07 | 0.68 | 0.09 | 0.23 |  |  |  |

***S3.5 Comparison of dielectric and wave transmission properties between the V-AE/BN-NH_2_ film and reported BN based materials***

| Ref. | dielectric constant | dielectric loss | wave transparency |
| --- | --- | --- | --- |
| [S29] | ε <3.8  (2~18 GHz) | tanδ <0.01  (2~18 GHz) | ∣T∣^2^﹥95%  (2~18 GHz) |
| [S30] | ε ≈ 0  (2~18 GHz) | Tanδ =0.001~0.1  (2~18 GHz) | ∣T∣^2^﹥96%  (2~18 GHz) |
| [S31] | ε =3~4  (1~12 MHz) | tanδ <0.001  (1~12 MHz) | / |
| [S32] | / | tanδ<0.0021~0.0024  (25C°) | / |
| [S33] | ε =2~3  (26.5 ~ 40 GHz) | tanδ < 0.3  (26.5 ~ 40 GHz) | / |
| This Work | ε < 4.3  (10^3^~10^5^Hz) | tanδ < 0.07  (10^3^~10^5^Hz) | ∣T∣^2^﹥99%  (2~18 GHz) |

**Supplementary References**

1. S. J. Clark, M. D. Segall, C. J. Pickard, P. J. Hasnip, M. J. Probert, First principles methods using castep. Z. Kristallogr. -Crystalline Materials **220**, 567–570 (2005). <https://doi.org/10.1524/zkri.220.5.567.65075>
2. B. K. Perdew J P, Ernzerhof M. Generalized gradient approximation made simple. Phys. Rev. Lett. 77(18), 3865–3868 (1996). <https://doi.org/10.1103/PhysRevLett.77.3865>
3. H.J. Monkhorst, J.D. Pack, Special points for Brillouin-zone integrations. Phys Rev B. **13**(12), 5188–5192 (1976). <https://doi.org/10.1103/PhysRevB.13.5188>
4. S. Grimme, J. Antony, S. Ehrlich, H. Krieg, A consistent and accurate *ab initio* parametrization of density functional dispersion correction (DFT-D) for the 94 elements H-Pu. J. Chem. Phys. **132**(15), 154104 (2010). <https://doi.org/10.1063/1.3382344>
5. K. Momma, F. Izumi, *VESTA 3*for three-dimensional visualization of crystal, volumetric and morphology data. J. Appl. Crystallogr. **44**(6), 1272–1276 (2011). <https://doi.org/10.1107/s0021889811038970>
6. X. Gu, L. Shaw, K. Gu, M.F. Toney, Z. Bao, The *Meniscus*-guided deposition of semiconducting polymers. Nat. Commun. **9**, 534 (2018). <https://doi.org/10.1038/s41467-018-02833-9>
7. K. Xu, Y. Wang, Z. Zhang, M. Li, R. Yang et al., Magnetic field-oriented one-step fabrication of metal-grade thermally conductive carbon fiber flexible thermal pad. Adv. Funct. Mater. **35**(43), 2505225 (2025). <https://doi.org/10.1002/adfm.202505225>
8. Y. Wang, Y. Chen, W. Dai, Z. Zhang, X. Kong et al., Anisotropic black phosphorene structural modulation for thermal storage and solar-thermal conversion. Small **19**(52), 2303933 (2023). <https://doi.org/10.1002/smll.202303933>
9. S. Plimpton, Fast parallel algorithms for short-range molecular dynamics. J. Computational Phys. **117**(1), 1-19 (1995). <https://doi.org/10.1006/jcph.1995.1039>
10. H. Ji, D.P. Sellan, M.T. Pettes, X. Kong, J. Ji et al., Enhanced thermal conductivity of phase change materials with ultrathin-graphite foams for thermal energy storage. Energy Environ. Sci. **7**(3), 1185–1192 (2014). <https://doi.org/10.1039/c3ee42573h>
11. Z. Wu, C. Xu, C. Ma, Z. Liu, H.-M. Cheng et al., Synergistic effect of aligned graphene nanosheets in graphene foam for high-performance thermally conductive composites. Adv. Mater. **31**(19), 1900199 (2019). <https://doi.org/10.1002/adma.201900199>
12. X. Shen, Z. Wang, Y. Wu, X. Liu, Y.-B. He et al., A three-dimensional multilayer graphene web for polymer nanocomposites with exceptional transport properties and fracture resistance. Mater. Horiz. **5**(2), 275–284 (2018). <https://doi.org/10.1039/c7mh00984d>
13. X. Xie, J. Wu, Y. Ma, S. Li, J. Yan, Low dielectric and high thermal conductive phononic crystal nanofiber metamaterial film. Adv. Mater. **37**(41), 2502146 (2025). <https://doi.org/10.1002/adma.202502146>
14. P. Liao, H. He, H. Guo, H. Niu, L. Kang et al., Highly thermally conductive boron nitride fiber. ACS Nano **19**(16), 16043–16052 (2025). <https://doi.org/10.1021/acsnano.5c02929>
15. W.-H. Han, Q.-Y. Wang, Y. Long, M. Xin, Y.-Z. Long et al., Personalized customization of in-plane thermal conductive networks by a novel electrospinning method. Compos. Part B Eng. **290**, 111971 (2025). <https://doi.org/10.1016/j.compositesb.2024.111971>
16. S. Miao, X. Wang, Z. Dong, Y. Xiang, L. Wang et al., Dopamine functionalized BNNS/PAN core-shell nanofiber membrane with high thermal conductivity and excellent dielectric properties. Compos. Part B Eng. **307**, 112909 (2025). <https://doi.org/10.1016/j.compositesb.2025.112909>
17. Z. Wang, L. Fan, R. Li, Y. Xu, Q. Fu, Preparation of polymer composites with high thermal conductivity by constructing a “double thermal conductive network” *via* electrostatic spinning. Compos. Commun. **36**, 101371 (2022). <https://doi.org/10.1016/j.coco.2022.101371>
18. Y. Liao, D. Wang, M. Ma, S. Chen, Y. Shi et al., Based on electrostatic adsorption constructing neural network-like structure of BNNS-OH@CNF composite films with excellent thermal management and electrical insulation performance. Compos. Part B Eng. **295**, 112197 (2025). <https://doi.org/10.1016/j.compositesb.2025.112197>
19. L.-H. Zhao, Y. Liao, L.-C. Jia, Z. Wang, X.-L. Huang et al., Ultra-robust thermoconductive films made from aramid nanofiber and boron nitride nanosheet for thermal management application. Polymers **13**(13), 2028 (2021). <https://doi.org/10.3390/polym13132028>
20. B. Zhu, Y. Qin, M. Li, Z. Zhang, Y. Wang et al., Thermal conductive radiative cooler enabled by Janus structure for above-ambient daytime cooling. Nano Energy **141**, 111124 (2025). <https://doi.org/10.1016/j.nanoen.2025.111124>
21. Z. Zheng, X. Gu et al., Electrically insulating yet excellent EMI shielding FeSiAl/CNF composite film with thermal conductivity for electronic packaging applications. ACS Appl. Mater. Interfaces **17**(15), 23176–23187 (2025). <https://doi.org/10.1021/acsami.5c01142>
22. J. Men, B. Xiang, X. Mao, W. Ren, Z. Yang et al., Ultra-flexible and mechanically strong silver nanowires/PBO nanofibers composite films for thermal management and photothermal conversion. J. Colloid Interface Sci. **700**, 138352 (2025). <https://doi.org/10.1016/j.jcis.2025.138352>
23. L. Tang, J. Jiang, Q. Liu, Q. Li, Q. Hu et al., A bioinspired, robust *Mica*/chitosan/PBO nanofiber paper with excellent dielectric insulation properties and high thermal conductivity. Ceram. Int. **51**(19), 29752–29761 (2025). <https://doi.org/10.1016/j.ceramint.2025.04.178>
24. Z. Miao, G. Zhu, L. Yang, P. Ding, F. Jiang, Multiple crosslinking-Stretch-induced multi-level orientation structure BNNS/Ca-SA/D films with high thermal conductive performance. Compos. Part B Eng. **309**, 113032 (2026). <https://doi.org/10.1016/j.compositesb.2025.113032>
25. J. Zhang, C. Lu, J. He, Bioinspired nacre-layered ANF/B-BNNS composite films with GN-bridged 3D thermal networks *via* scalable sol-gel fabrication. J. Alloys Compd. **1038**, 182529 (2025). <https://doi.org/10.1016/j.jallcom.2025.182529>
26. Q. Chen, J. Feng, Y. Xue, S. Huo, T. Dinh et al., An engineered heterostructured trinity enables fire-safe, thermally conductive polymer nanocomposite films with low dielectric loss. Nano-Micro Lett. **17**(1), 168 (2025). <https://doi.org/10.1007/s40820-025-01681-9>
27. N. Li, Z. Lu, D. Ning, L. Hua, S. E, Mechanically strong heterocyclic aramid nanofiber-based nanopaper *via* boron nitride nanosheet-incorporated polymerization and assembly of poly(p-phenylene-benzimidazole-terephthalamide). Chem. Eng. J. **521**, 166648 (2025). <https://doi.org/10.1016/j.cej.2025.166648>
28. J. Zhang, X. Kong, Y. Wang, Z. Zhang, L. Li et al., Recycled and flexible boron nitride heat spread film with high thermal conductivity. J. Mater. Chem. C **11**(39), 13204–13212 (2023). <https://doi.org/10.1039/d3tc02761a>
29. Z. Chen, B. Li, H. Tang, Y. Zhang, S. Wen et al., Synergistic enhancement of the performance of BN based wave-transparent composites by hybrid weaving of Si_3_N_4_ and SiO_2_ fibers. Compos. Part A Appl. Sci. Manuf. **208**, 109897 (2026). <https://doi.org/10.1016/j.compositesa.2026.109897>
30. Z. Xie, Y. Tang, Z. Luo, Y. Zhang, W. Zheng et al., Dual metal synergistic modulation of boron nitride for high-temperature wave-transparent metamaterials. Mater. Horiz. **12**(5), 1547–1557 (2025). <https://doi.org/10.1039/d4mh01020e>
31. Y. Jing, A.V. Korovina, J. Xie, J. Xia, B. Li et al., Fixing disordered hexagonal boron nitride grains leads to simultaneous high strength and deformability. Adv. Funct. Mater. **35**(51), e08767 (2025). <https://doi.org/10.1002/adfm.202508767>
32. B. Niu, D. Cai, Z. Yang, X. Duan, W. Duan et al., Reducing high-temperature dielectric loss of h-BN ceramics by orienting grains. Scr. Mater. **223**, 115098 (2023). <https://doi.org/10.1016/j.scriptamat.2022.115098>
33. W. Qiao, J. Yang, J. Qiao, H. Gao, Z. Li et al., Pressureless-sintered boron nitride nanosheets/glass composite ceramics for excellent mechanical, dielectric and thermo-conductive performances. J. Eur. Ceram. Soc. **43**(9), 3998–4007 (2023). <https://doi.org/10.1016/j.jeurceramsoc.2023.02.072>
